# FABP4‐mediated lipid droplet accumulation drives epithelial–mesenchymal transition and aggravates alveolar epithelial barrier disruption

**DOI:** 10.1002/ctm2.70563

**Published:** 2025-12-26

**Authors:** Zihao Shen, Yuanpu Qi, Mingyu Chu, Minchao Wu, Chen Feng, Xiangyu Li, Zhaoyang Liu, Linjie Si, Yongliang Wang, Jialin Zhang, Xiaoning Lu, Peng Lu

**Affiliations:** ^1^ Department of Cardiovascular Surgery The First Affiliated Hospital with Nanjing Medical University Nanjing Jiangsu China; ^2^ Department of Cardiothoracic Surgery The Affiliated Suqian First People's Hospital of Nanjing Medical University Suqian Jiangsu China

**Keywords:** alveolar epithelial barrier, epithelial–mesenchymal transition, FABP4, lipid droplets, lung ischemia/reperfusion injury

## Abstract

**Background:**

Acute respiratory distress syndrome (ARDS) frequently develops after cardiopulmonary bypass (CPB), with lung ischemia/reperfusion injury (LIRI) as a major contributing factor. However, the role of fatty acid‐binding protein 4 (FABP4) in the pathogenesis of CPB‐associated ARDS remains poorly understood.

**Methods:**

Experimental LIRI models were established in vivo and in vitro to investigate the role of FABP4 in alveolar epithelial injury. Lipid droplets (LDs) accumulation, fatty acid (FA) metabolism, epithelial‐mesenchymal transition (EMT), and alveolar epithelial barrier (AEB) integrity were assessed using molecular, cellular, and functional approaches. Pharmacological and genetic interventions were applied to evaluate the contribution of FABP4‐mediated signaling pathways.

**Results:**

LIRI induced autocrine FABP4 signaling in alveolar epithelial cells, leading to pronounced LDs accumulation and disruption of AEB integrity. FABP4 activation enhanced FA metabolism and promoted EMT, which played a critical role in epithelial barrier dysfunction. Mechanistically, FABP4 activated the p38 MAPK pathway, resulting in ULK1 phosphorylation, suppression of lipophagy, and subsequent LDs formation, thereby driving EMT. Inhibition of LDs accumulation effectively attenuated EMT and alleviated AEB disruption.

**Conclusion:**

FABP4 serves as a key metabolic regulator linking lipid reprogramming to EMT and alveolar epithelial barrier disruption during LIRI. Targeting FABP4‐mediated lipid metabolism may represent a promising therapeutic strategy for preventing ARDS following CPB.

**Key points:**

- LIRI induces autocrine FABP4 signaling in alveolar epithelial cells.
- FABP4 promotes lipid droplets accumulation by inhibiting lipophagy through p38 MAPKULK1 signaling.
- FABP4‐driven lipid metabolic reprogramming triggers EMT and disrupts alveolar epithelial barrier integrity.
- Targeting FABP4 or lipid droplets accumulation may offer therapeutic potential for CPB‐associated ARDS.

## Graphical abstract

- LIRI induces FABP4 secretion, driving lipid droplets accumulation in alveolar epithelial cells.
- FABP4 activates p38 MAPK/ULK1 signalling to inhibit lipophagy and promote lipid storage.
- Lipid droplets trigger epithelial–mesenchymal transition and disrupt alveolar barrier.
- Elevated FABP4 predicts ARDS in cardiopulmonary bypass patients.

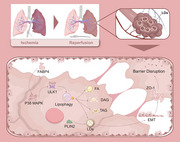

## 1 INTRODUCTION

Acute respiratory distress syndrome (ARDS)—defined by bilateral lung infiltrates and severe hypoxemia from non‐cardiac pulmonary oedema—frequently complicates cardiopulmonary bypass (CPB) procedures.^1^, ^2^ ARDS is associated with significant in‐hospital and long‐term morbidity and mortality, as well as prolonged hospitalisation.^3^ Among the leading causes of early lung injury and subsequent ARDS after CPB is lung ischaemia/reperfusion injury (LIRI).^4^ LIRI disrupts intercellular junctions among alveolar epithelial cells, promotes pulmonary leukocyte infiltration and compromises the alveolar epithelial barrier (AEB), thereby contributing to the development of ARDS.^5^ Despite its clinical relevance, the molecular mechanisms underlying LIRI remain poorly understood. Furthermore, decades of research have yet to yield effective pharmacologic therapies for ARDS, and neither mechanical ventilation nor evidence‐based supportive care offer meaningful protection.^6^, ^7^

Recent studies, including our own, have indicated that elevated serum free fatty acid (FFA) levels may serve as predictive markers for ARDS following cardiac surgery.^8^ Our lipidomic analyses further revealed that fatty acid (FA) metabolism is significantly altered in response to LIRI, with marked lipid droplets (LDs) accumulation observed in alveolar epithelial cells. While LDs formation in alveolar macrophages has emerged as a hallmark of pulmonary inflammation,^9^ the role of epithelial LDs accumulation has been largely overlooked. Previous studies have shown that inhibiting LDs formation can reduce viral replication and inflammation in influenza‐infected macrophages.^10^, ^11^ Similarly, in ischaemic brain injury models, LDs accumulation in microglia promotes disease progression.^12^, ^13^ LDs accumulation has also been observed in bleomycin‐induced lung injury and in EGFR‐TKI‐resistant human lung cancers.^14^, ^15^ However, the dynamic changes and pathological roles of epithelial LDs accumulation and lipophagy in lung injury remain insufficiently characterised. Intriguingly, inhibition of lipophagy can preserve LDs integrity and attenuate damage in myocardial ischaemia/reperfusion injury,^16^ suggesting that LIRI may significantly reshape alveolar epithelial metabolic phenotypes.

FA‐binding protein 4 (FABP4), a member of the FABP family, plays a pivotal role in lipid metabolism and inflammation.^17^ FABP4 expression positively correlates with circulating FFA levels, and its inhibition has been shown to reduce inflammation and apoptosis in epithelial cells.^18^ Injured epithelial cells up‐regulate FABP4, which in turn drives LDs accumulation and enhances FA oxidation.^19^ In cancer, FABP4 acts as a lipid mediator that promotes unsaturated FFA‐induced lipid accumulation and facilitates breast cancer metastasis.^20^ However, whether FABP4 contributes to LDs accumulation and AEB disruption during lung injury remains largely unexplored. FABP4 has been implicated in epithelial–mesenchymal transition (EMT) through CD36 up‐regulation in glioma cells,^21^ and its inhibition can suppress adipocyte‐induced cancer cell metastasis and EMT phenotypes.^22^ Additionally, FABP4 overexpression activates the p38 MAPK pathway, promoting pro‐inflammatory, oxidative and apoptotic responses in epithelial cells.^23^ The MAPK signalling axis is a well‐established driver of persistent EMT and invasion in multiple cancers,^24^, ^25^ and FABP4 silencing has been shown to reduce ER stress‐induced apoptosis in myocardial ischaemia/reperfusion models.^26^ These findings underscore the multifaceted role of FABP4 in regulating lipid metabolism, inflammation and cellular plasticity.

In this study, we identified LDs accumulation as a prominent feature of hypoxia/reoxygenation (HR)‐stimulated alveolar epithelial cells and a key driver of EMT. Single‐cell transcriptomic analysis revealed FABP4 as a consistently up‐regulated transcript in epithelial cells subjected to LIRI. Building on this observation, we demonstrate how FABP4‐mediated lipid metabolic reprogramming promotes EMT and compromises AEB integrity. In particular, we show that autocrine FABP4 signalling facilitates LDs formation, which serves as an energy source fuelling barrier disruption, fibrotic remodelling, and impaired tissue homeostasis.

## 2 RESULTS

### 2.1 Alveolar epithelial cells accumulate LDs during LIRI

**FIGURE 1:**
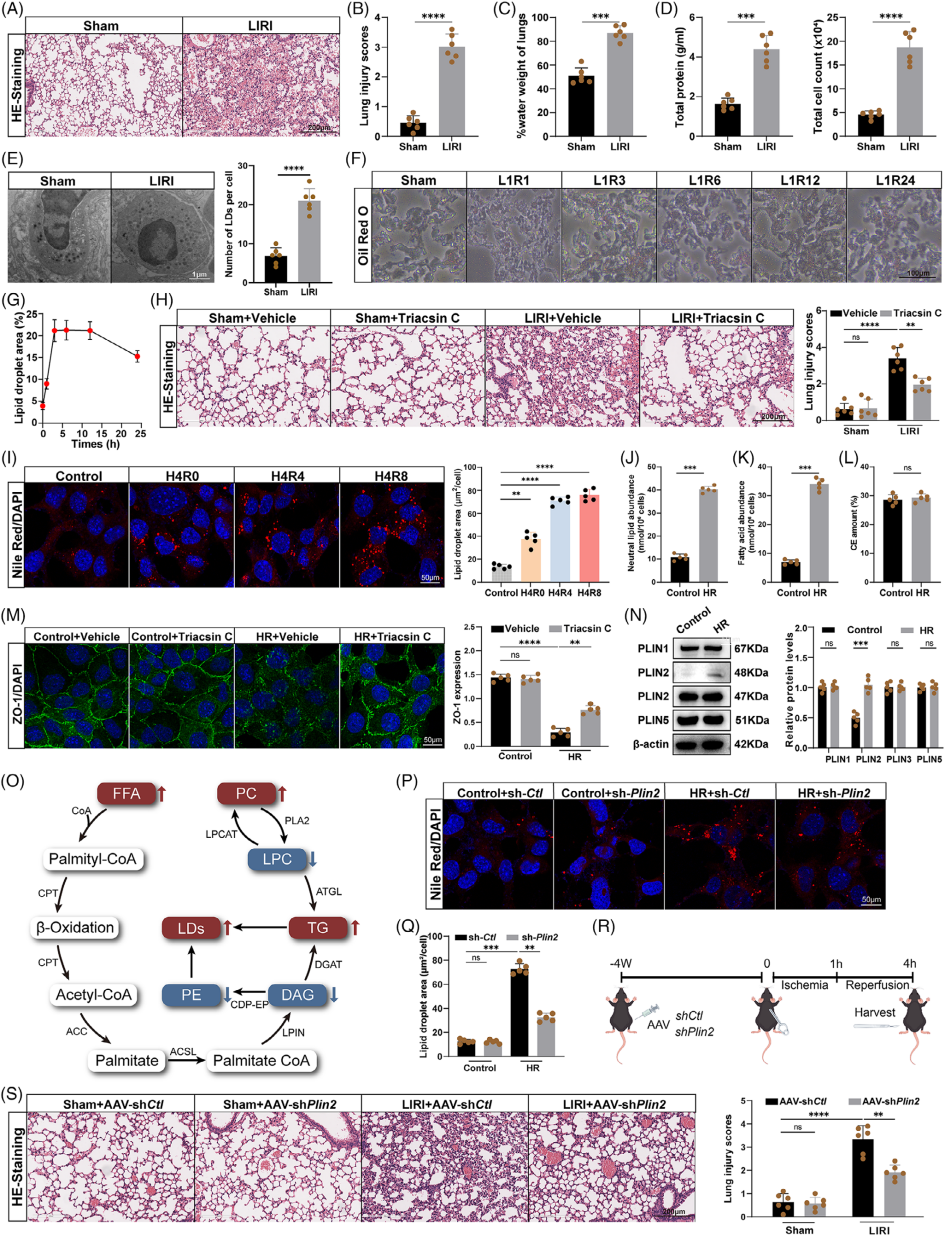
Alveolar epithelial cells accumulate lipid droplets in LIRI. (A and B) HE staining of lung sections and corresponding lung injury scores (*n* = 6). Scale bars: 200 µm. (C) Lung wet/dry weight ratio (*n* = 6). (D) Total protein content and cell count in BALF (*n* = 6). (E) Electron microscopy of LDs in mice lung (*n* = 6). Scale bars = 1 µm. (F and G) Oil Red O staining of mouse lung at different reperfusion time points (sham, 1, 3, 6, 12, and 24 h after 1 h ischaemia) and quantification of staining intensity (*n* = 6). (H) HE staining and lung injury scores in mice treated with vehicle or triacsin C (2 mg/kg, i.p.) before LIRI. Triacsin C significantly reduced lung injury. Scale bars: 200 µm (*n* = 6). (I) Nile red staining of MLE‐12 cells under Control, H4R0 (4 h hypoxia + 0 h reoxygenation), H4R4 (4 h hypoxia + 4 h reoxygenation), H4R8 (4 h hypoxia + 8 h reoxygenation) and quantification of staining intensity (*n* = 5). Scale bars: 50 µm. (J–L) Abundance of the total pool of neutral lipids (*n* = 5). (J) neutral lipids, (K) FA, (L) cholesteryl esters in MLE‐12 cells under HR. (M) Representative immunofluorescence images of ZO‐1 (green) and DAPI (blue) in MLE‐12 cells treated with vehicle or triacsin C (5 µM) under normoxia or HR conditions. Quantification of ZO‐1 fluorescence intensity is shown on the right. Scale bars = 50 µm. (N) The protein expression of PLIN1, PLIN2, PLIN3, PLIN5 in MLE‐12 cells under HR conditions, normalised to β‐actin (*n* = 5). (O) Scheme depicting the pathways leading to lipid droplet biogenesis (red: increased, blue: decreased). (P and Q) Nile red staining of MLE‐12 cells model of sh*‐Plin2* under HR and quantification of staining intensity (*n* = 5). Scale bars: 50 µm. (R) Schematic illustration of the in vivo injection of PLIN2‐knockdown AAV (sh*Plin2*), and knockdown control AAV (sh*Ctl*) into mouse 4 weeks before LIRI. (S) HE staining of the LIRI mouse model of AAV‐sh*Plin2* and corresponding lung injury scores (*n* = 6). Scale bars: 200 µm. All data are presented as means ± SD. Statistical significance was determined by unpaired Student's *t*‐test or one‐way ANOVA followed by Tukey's post hoc test. ns: not significant, **p < .01, ***p < .001, ****p < .0001.

Building on our previous findings on FFA metabolism, we investigated intracellular LDs formation in alveolar epithelial cells during LIRI. We employed a murine hilar clamp model (1‐h ischaemia followed by 3‐h reperfusion) as previously described.^27^ The LIRI phenotype was validated by histological assessment (H&E staining), increased lung wet‐to‐dry weight ratio, elevated protein concentration and total cell count in bronchoalveolar lavage fluid (BALF) (Figure 1A–D). Transmission electron microscopy revealed spherical, electron‐lucent structures in alveolar epithelial cells, later identified as LDs (Figure 1E). Oil Red O staining at multiple reperfusion time points (1, 3, 6, 12 and 24 h) showed that LDs accumulation peaked at 1–3 h and gradually declined thereafter (Figure 1F). To model this in vitro, we subjected MLE‐12 cells to HR, which led to a time‐dependent increase in LDs as revealed by Nile Red staining (Figure 1G). Lipidomics indicated an increase in neutral lipids, primarily FFAs and triglycerides (TGs), but not cholesteryl esters (CEs) (Figure 1J–L). To validate the causal role of LDs, we used triacsin C, an LDs formation inhibitor. Triacsin C reduced LIRI‐induced lung injury in mice (Figure 1H) and preserved zonula occludens‐1 (ZO‐1) expression in HR‐treated cells (Figure 1M), demonstrating that LDs accumulation causally contributes to barrier dysfunction and lung injury. These findings were corroborated by comprehensive lipidomic analysis in LIRI mouse lungs (Figure 1O).

We next sought to identify key regulators of LDs biogenesis in HR‐exposed epithelial cells. Among the perilipin (PLIN) family, only PLIN2 showed consistent up‐regulation at both mRNA and protein levels in HR‐treated MLE‐12 cells (Figures 1N and S1A). Knockdown of PLIN2 significantly reduced LDs accumulation (Figures 1P,Q and S1B). To study the in vivo relevance, we generated a high‐titer adeno‐associated virus (AAV) expressing epithelial‐specific shRNA targeting PLIN2 (AAV‐sh*Plin2*) or a scrambled control (AAV‐sh*Ctl*), which was delivered via tail vein injection. Two independent shRNA sequences targeting PLIN2 both effectively reduced PLIN2 expression under baseline conditions (Figure S1C). Upon LIRI challenge, mice treated with AAV‐sh*Plin2* showed reduced lung injury, as evidenced by improved histology, lower lung wet‐to‐dry ratio, and decreased BALF protein content and cell count (Figures 1R,S and S1D–F). Collectively, these results demonstrate that LIRI induces LD accumulation in alveolar epithelial cells, with PLIN2 playing a critical role in mediating LDs biogenesis and contributing to lung injury.

### 2.2 LDs promote disruption of AEB

**FIGURE 2:**
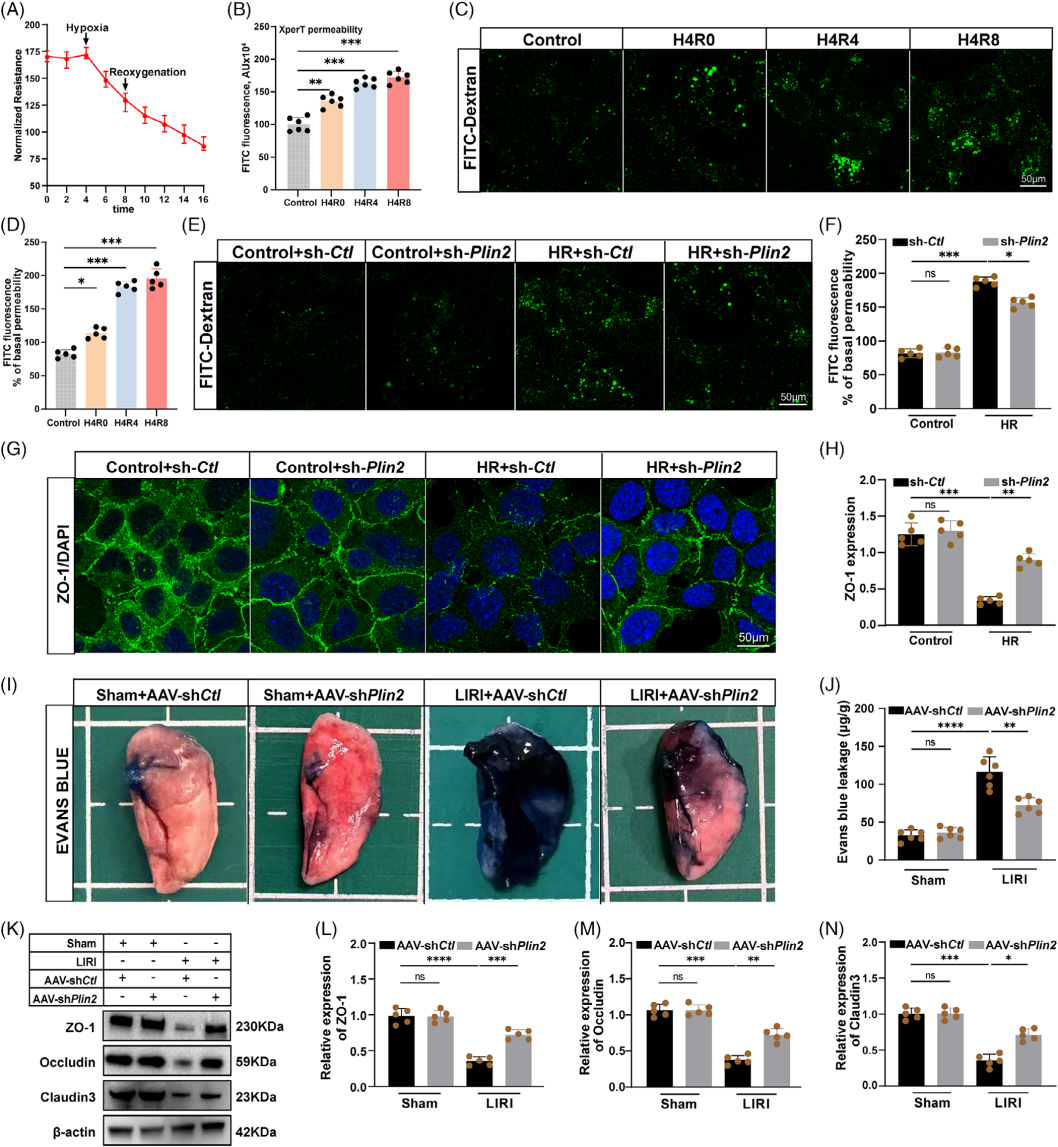
Lipid droplets support the disruption of alveolar epithelial barrier. (A) MLE‐12 cells plated on microelectrodes were measured with trans‐epithelial electric resistance (TEER) under hypoxia and then stimulated with reoxygenation (marked by arrow) (*n* = 5). (B) Analysis of MLE‐12 cells permeability for macromolecules using fluorescein isothiocyanate (FITC)‐labelled avidin as a tracer (*n* = 5). (C and D) Visualisation of FITC–avidin accumulation underneath MLE‐12 cells monolayers reflecting HR‐induced MLE‐12 barrier dysfunction (*n* = 5). Scale bars = 50 µm. (E–H) MLE‐12 cells were infected with a PLIN2‐knockdown lentivirus (sh*‐Plin2*) and a knockdown control lentivirus (sh‐*Ctl*). (E and F) Visualisation of FITC–avidin accumulation underneath MLE‐12 cells monolayers reflecting HR‐induced MLE‐12 barrier dysfunction (*n* = 5). Scale bars = 50 µm. (G and H) Tight junctions were visualised by staining for ZO‐1 (*n* = 5). Scale bars = 50 µm. (I–N) Mice were injected with PLIN2‐knockdown AAV (sh*Plin2*) *and* knockdown control AAV (sh*Ctl*). (I and J) Representative images and quantification of Evans blue dye extravasation in lung tissue (*n* = 6). (K–N) The protein expression of ZO‐1, occludin, claudin‐3 in mice under LIRI, normalised to β‐actin (*n* = 5). All data are presented as means ± SD. Statistical significance was determined by unpaired Student's *t*‐test or one‐way ANOVA followed by Tukey's post hoc test. ns: not significant, *p < .05, **p < .01, ***p < .001, ****p < .0001.

To investigate the role of LDs in HR‐induced alveolar epithelial injury, we assessed AEB function in MLE‐12 cells. Trans‐epithelial electrical resistance (TEER) measurements showed a significant reduction in barrier integrity following HR exposure (Figure 2A). Permeability assays using fluorescein isothiocyanate (FITC)–dextran revealed increased epithelial permeability under HR conditions (Figure 2B). Visualisation of FITC–dextran penetration confirmed enhanced barrier leakage, indicated by intensified green fluorescence in the substrate beneath the epithelial monolayer (Figure 2C,D). To determine the contribution of LDs to this barrier disruption, we knocked down PLIN2, a key regulator of LDs formation. PLIN2 silencing significantly attenuated FITC–dextran permeability under HR, indicating improved barrier integrity (Figure 2E,F). Given the essential role of tight junctions in maintaining epithelial barrier function, we evaluated the expression of ZO‐1, a critical tight junction protein. HR exposure resulted in ZO‐1 down‐regulation and junctional disruption, while PLIN2 knockdown restored ZO‐1 expression and preserved cell–cell contacts (Figure 2G,H).

Next, we extended these findings in vivo using our LIRI model. Evans blue dye extravasation revealed that AAV‐shPlin2 significantly reduced pulmonary vascular leakage (Figure 2I,J). Western blot analysis of tight junction proteins further supported these observations: LIRI markedly decreased the expression of ZO‐1, occludin, and claudin‐3 in AAV‐shCtl lungs, whereas PLIN2 knockdown preserved their expression (Figure 2K–N). Taken together, these data demonstrate that PLIN2‐mediated LDs accumulation compromises AEB integrity, and that inhibition of LDs formation mitigates HR‐ and LIRI‐induced epithelial barrier disruption.

### 2.3 FABP4 signalling drives LDs formation and AEB disruption

**FIGURE 3:**
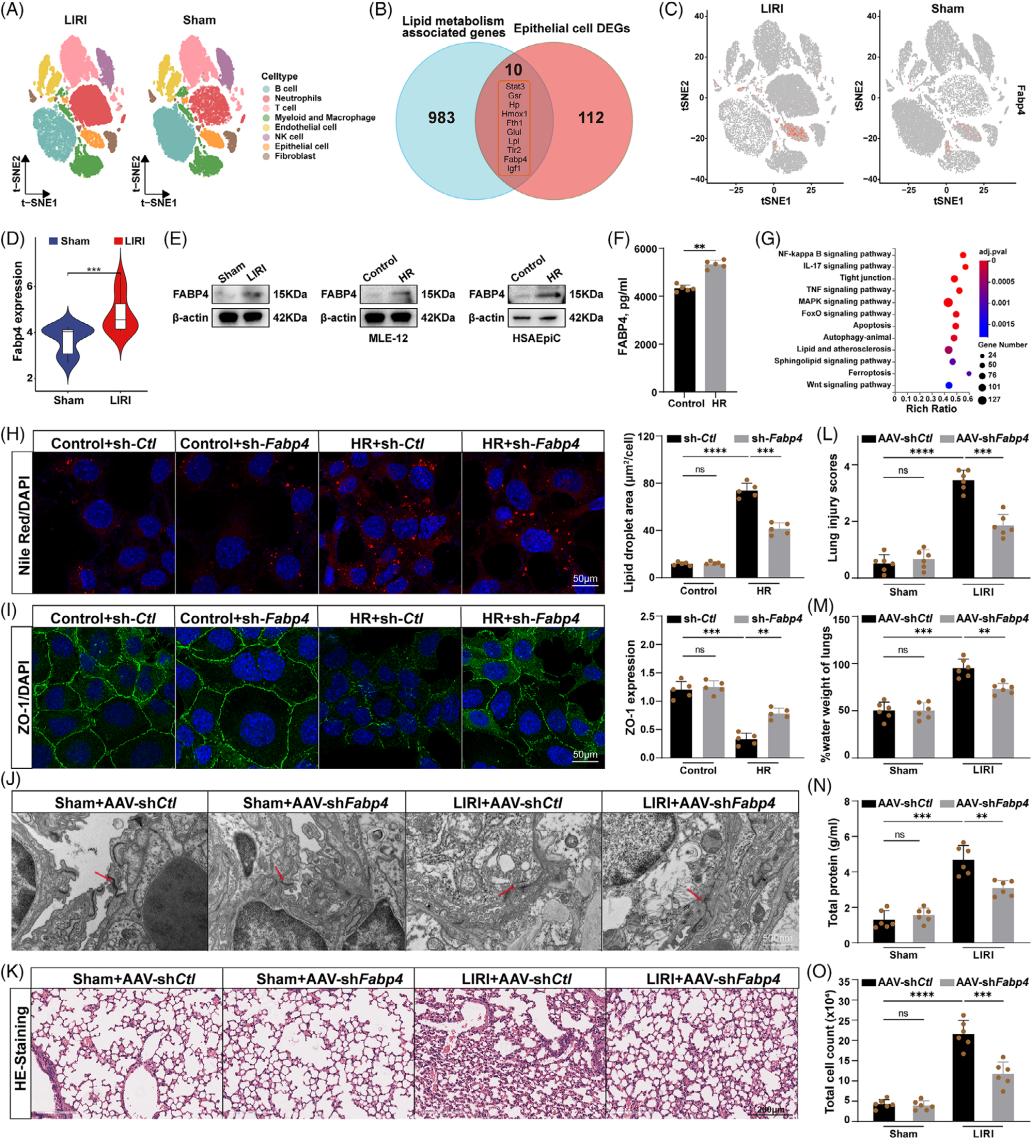
FABP4 signalling supports LDs formation and alveolar epithelial barrier disruption. (A) tSNE plot all scRNA‐seq cells colour‐coded by cell type comparing LIRI and sham groups. (B) Venn diagram showing overlap between differentially expressed genes (DEGs) in epithelial cells and lipid metabolism associated genes. (C and D) tSNE and violin plot showing the expression of Fabp4 in epithelial cells from the lung of LIRI and sham groups. (E) The protein expression of FABP4 in MLE‐12 and HSAEpiC under HR and mice under LIRI, normalised to β‐actin (*n* = 5). (F) Corresponding extracellular media of FABP4 in MLE‐12 cells under HR (*n* = 5). G) Bubble plot of enriched KEGG pathways for DEGs in LIRI and sham groups. (H and I) MLE‐12 cells were infected with a FABP4‐knockdown lentivirus (*sh‐Fabp4*) and a knockdown control lentivirus (sh‐*Ctl*). (H) Nile red staining of MLE‐12 cells model of sh*‐Fabp4* under HR and quantification of staining intensity (*n* = 5). Scale bars = 50 µm. (I) Tight junctions were visualised by staining for ZO‐1 (*n* = 5). Scale bars = 50 µm. (J–O) Mice were injected with FABP4‐knockdown AAV (sh*Fabp4*) *and* knockdown control AAV (sh*Ctl*). (J) Representative transmission electron micrographs of the ultrastructure of the LIRI mouse model of AAV‐sh*Fabp4* (*n* = 6). Scale bars = 500 nm. (K and L) HE staining of lung sections and corresponding lung injury scores (*n* = 6). Scale bars: 200 µm. (M) Lung wet/dry weight ratio (*n* = 6). (N and O) Total protein content and cell count in BALF (*n* = 6). All data are presented as means ± SD. Statistical significance was determined by unpaired Student's *t*‐test or one‐way ANOVA followed by Tukey's post hoc test. ns: not significant, *p <.05, **p < .01, ***p < .001, ****p < .0001.

To explore the upstream mechanisms regulating LDs accumulation and epithelial barrier disruption, we performed single‐cell RNA sequencing (scRNA‐seq) on lung tissues from LIRI mice. A total of 44 152 cells were analysed and clustered into distinct cell types based on established markers from the LungMAP and ImmGen databases. These included epithelial cells, endothelial cells, macrophages, myeloid cells, B cells, T cells, NK cells, neutrophils and fibroblasts (Figure 3A). Within the epithelial cell population, we identified 77 significantly up‐regulated and 45 down‐regulated genes in LIRI compared with sham (Figure S2A). Among the top 10 lipid metabolism‐related transcripts, we identified *Fabp4*, encoding FABP4, a cytoplasmic chaperone known for its roles in FA uptake, transport and metabolism (Figure 3B–D). Among the top lipid metabolism‐related transcripts identified by scRNA‐seq, we focused on FABP4 based on its well‐established role in FA uptake and LDs formation, its documented involvement in inflammatory responses, and the availability of pharmacological inhibitors with translational potential. FABP4 mRNA was significantly increased in both LIRI lung tissue and HR‐exposed MLE‐12 cells, as confirmed by qPCR (Figure S2B). Protein analysis further demonstrated increased intracellular and secreted levels of active FABP4 under HR conditions and in LIRI lungs (Figures 3E,F and S2C–F). To validate these findings in human cells, we performed HR treatment on human small airway epithelial cells (HSAEpiC) and observed a similar up‐regulation of FABP4 expression, consistent with our findings in MLE‐12 cells (Figure 3E). FABP4 expression in lung tissue peaked at 1–3 h post‐reperfusion, mirroring the temporal pattern of LDs accumulation (Figure S2G). Furthermore, to investigate the mechanism driving this up‐regulation, bulk RNA‐seq analysis indicated activation of MAPK and tight junction pathways downstream of FABP4 signalling (Figure 3G). Functionally, FABP4 knockdown in HR‐stimulated MLE‐12 cells significantly reduced LDs accumulation (Figures 3H and S2H) and restored ZO‐1 expression (Figure 3I), suggesting a protective effect on barrier integrity. To assess these effects in vivo, we administered epithelial‐specific AAV‐sh*Fabp4*. Two independent shRNA sequences targeting FABP4 both effectively reduced FABP4 expression (Figure S2I,J) and preserved alveolar ultrastructure, as shown by electron microscopy (Figure 3J). Furthermore, FABP4 knockdown significantly attenuated alveolar epithelial injury and pulmonary permeability in LIRI mice (Figure 3K–O). ELISA analysis showed that AAV‐sh*Fabp4* significantly reduced LIRI‐induced elevation of pro‐inflammatory cytokines IL‐6 and TNF‐α in lung tissue (Figure S2K,L), demonstrating that FABP4 promotes inflammatory responses during LIRI. Importantly, treatment with a MAPK agonist reversed the protective effects of FABP4 knockdown, restoring LDs accumulation and barrier dysfunction (Figure S2M,N), highlighting the importance of FABP4–MAPK signalling in this process. Collectively, these findings demonstrate that FABP4 promotes LDs formation and contributes to AEB disruption through activation of MAPK signalling.

### 2.4 FABP4‐mediated lipophagy promotes FA metabolism and TG formation

**FIGURE 4:**
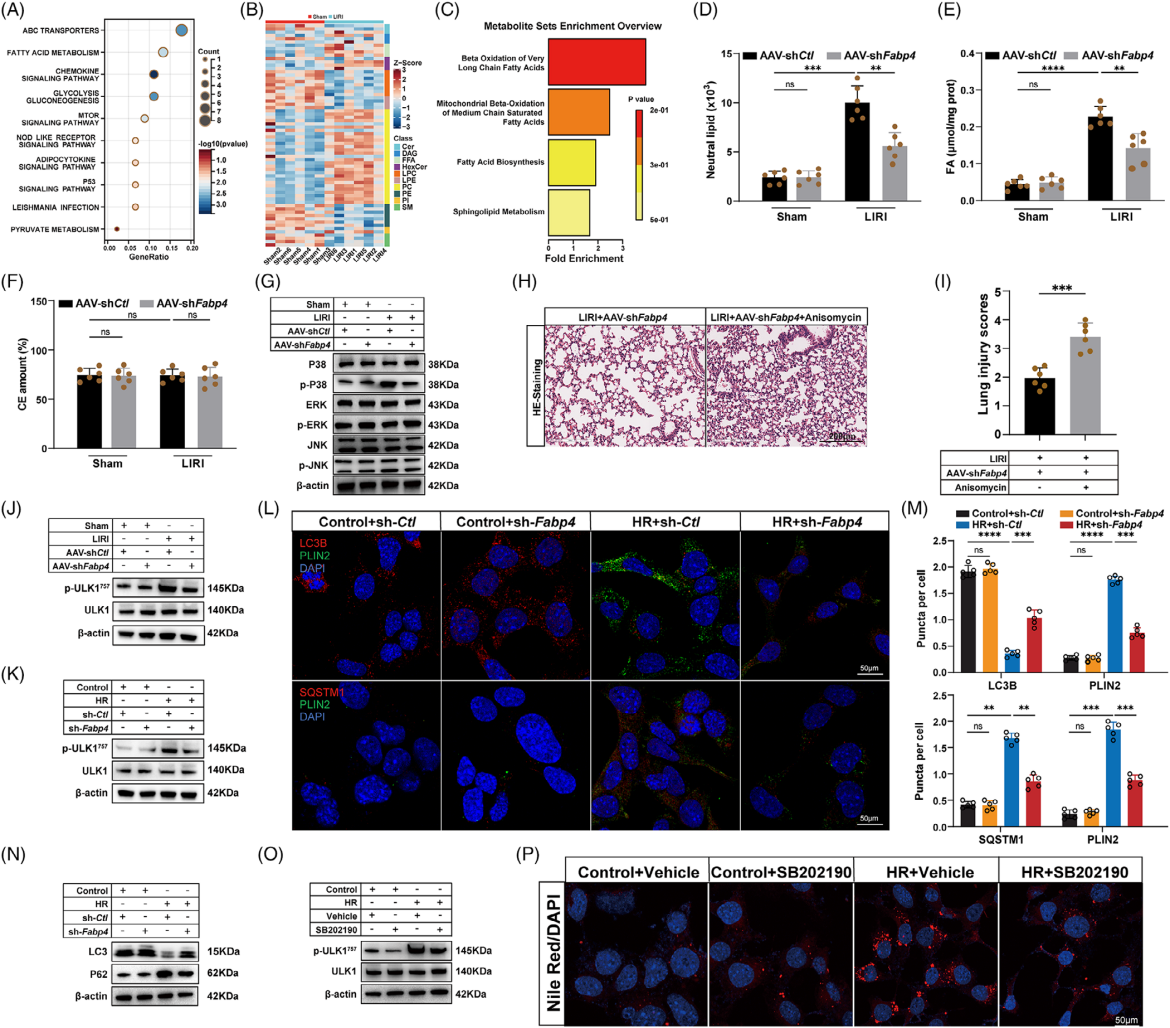
FABP4‐mediated lipophagy promotes FFA metabolism and TG formation. (A) GSEA enrichment of differentially expressed genes (DEGs) in epithelial cells between LIRI and sham groups. (B) Heatmap of differential lipid metabolites between LIRI and sham groups. (C) Metabolite set enrichment overview of differential metabolites between LIRI and sham groups. (D–J) Mice were injected with FABP4‐knockdown AAV (sh*Fabp4*) *and* knockdown control AAV (sh*Ctl*). (D–F) Abundance of the total pool of neutral lipids (*n* = 6). (D) Neutral lipids, (E) FA, (F) cholesteryl esters in mice under LIRI. (G) The protein expression of p38, p‐P38, ERK, p‐ERK, JNK, p‐JNK in mice under LIRI, normalised to β‐actin (*n* = 5). (H and I) HE staining of lung sections and corresponding lung injury scores in LIRI mice treated with AAV‐sh*Fabp4* and anisomycin (*n* = 6). Scale bars: 200 µm. (J) The protein expression of p‐ULK1^757^, ULK1 in mice under LIRI, normalised to β‐actin (*n* = 5). (K–N) MLE‐12 cells were infected with a FABP4‐knockdown lentivirus (sh*‐Fabp4*) and a knockdown control lentivirus (sh‐*Ctl*). (K) The protein expression of p‐ULK1^757^, ULK1 in MLE‐12 cells under HR, normalised to β‐actin (*n* = 5). (L and M) Confocal images of MLE‐12 cells treated with HR. Lipophagy was both visualised using LC3B and SQSTM1, and LDs were visualised using PLIN2. The number of LC3B puncta, SQSTM1 puncta and PLIN2 puncta were quantified (*n* = 5). Scale bars: 50 µm. (N) The protein expression of LC3‐II/LC3‐I ratio and P62 in MLE‐12 cells under HR, normalised to β‐actin (*n* = 5). (O and P) MLE‐12 cells were treated with p38 inhibitor SB202190 (10 µM). (O) The protein expression of p‐ULK1^757^, ULK1 in MLE‐12 cells under HR, normalised to β‐actin (*n* = 5). (P) Nile Red staining of MLE‐12 cells treated with SB202190 under HR (*n* = 5). Scale bars = 50 µm. All data are presented as means ± SD. Statistical significance was determined by unpaired Student's *t*‐test or one‐way ANOVA followed by Tukey's post hoc test. ns: not significant, **p < .01, ***p < .001, ****p < .0001.

To investigate the role of FABP4 in pulmonary metabolism, we focused on the epithelial cell cluster from our scRNA‐seq dataset. Gene set enrichment analysis (GSEA) of differentially expressed genes (DEGs) between LIRI and sham groups revealed significant enrichment in FA metabolism pathways (Figure 4A). Given the well‐established relationship between FA metabolism and FABP4, we next performed lipidomic analysis to explore the underlying mechanisms. Differential lipid metabolite analysis between LIRI and sham lung tissues identified several significant alterations (Table S1), which were visualised in a volcano plot (Figure S3A). A heatmap further demonstrated the clustering and expression patterns of these metabolites (Figure 4B). Kyoto Encyclopedia of Genes and Genomes (KEGG) pathway enrichment analysis highlighted dysregulation in FA metabolism as a potential key contributor to LIRI‐induced injury (Figure 4C). As expected, lipidomic analysis revealed a net increase in neutral lipids, particularly in the form of FAs, in LIRI mouse lung tissue (Figure 4D). A similar accumulation was observed in serum samples from LIRI mice (Figure S3B). Notably, the decrease in FABP4 expression in LIRI mice via AAV‐sh*Fabp4* significantly reduced the concentrations of FAs (Figures 4E,F and S3B), confirming the crucial role of FABP4 in regulating FA levels during LIRI.

We next examined whether MAPK signalling, identified as an upstream regulator of FABP4‐mediated LDs accumulation, also affects FA metabolism. While no changes were observed in total MAPK protein expression, we detected significant increases in p38 MAPK phosphorylation in LIRI lung tissues (Figures 4G and S3C). In contrast, AAV‐sh*Fabp4* treatment decreased p38 MAPK phosphorylation levels, whereas the phosphorylation status of ERK and JNK MAPKs remained unchanged (Figure S3D,E), indicating that the FABP4‐mediated effect is specifically mediated through the p38 MAPK pathway rather than other MAPK family members. Furthermore, co‐immunoprecipitation (Co‐IP) experiments in MLE‐12 cells confirmed that FABP4 physically interacts with p38 MAPK in HR‐treated cells (Figure S3F). To validate the functional importance of p38 MAPK in vivo, we treated AAV‐shFabp4 mice with anisomycin. Anisomycin reversed the protective effects of FABP4 knockdown (Figures 4H,I and S3G–I), confirming that p38 MAPK is essential for FABP4‐mediated lung injury.

Given the role of p38 MAPK in regulating ULK1, a key regulator of autophagy and lipophagy, we hypothesised that FABP4‐activated p38 MAPK promotes ULK1 phosphorylation at Ser757, an inhibitory site that suppresses autophagy. Indeed, we observed a significant up‐regulation of ULK1 phosphorylation (Ser757) in both LIRI lung tissues and HR‐stimulated MLE‐12 cells (Figures 4J,K and S3J,K). This up‐regulation was prevented by FABP4 knockdown, suggesting a role for FABP4 in regulating ULK1 activation. To confirm target specificity, we performed rescue experiments using recombinant FABP4 protein. Re‐expression of FABP4 in sh‐F*abp4* cells restored p38 phosphorylation and ULK1 phosphorylation (Figure S3L,M), confirming that these effects are specifically mediated by FABP4. Further experiments demonstrated that HR treatment in MLE‐12 cells led to a significant increase in PLIN2^+^ cells, a marker of LD formation, along with a decrease in LC3B puncta (an indicator of autophagosome formation) and an increase in SQSTM1 puncta (a marker of autophagic degradation) (Figure 4L,M), consistent with suppressed lipophagy. However, FABP4 knockdown reversed these changes, promoting the formation of LC3B puncta and reducing SQSTM1 puncta, indicating enhanced lipophagy. Western blot confirmed that HR decreased the LC3‐II/LC3‐I ratio and increased p62 accumulation, which were restored by FABP4 knockdown (Figures 4N and S3P). Additionally, triacsin C treatment independently increased the LC3‐II/LC3‐I ratio and decreased p62 under HR conditions (Figure S3Q), demonstrating that LD reduction promotes autophagy even without FABP4 manipulation. To further validate these findings, we treated MLE‐12 cells with SB202190, a p38 MAPK inhibitor. This treatment not only decreased ULK1 phosphorylation and promoted lipophagy, but it also reduced LDs accumulation, providing strong evidence for the involvement of p38 MAPK in FABP4‐mediated LDs formation (Figures 4O,P, S3N,O and S3R,S). In summary, these data suggest that FABP4 promotes LDs accumulation through p38 MAPK‐dependent lipophagy, which regulates FA metabolism and contributes to TG formation during LIRI.

### 2.5 EMT is regulated by FABP4‐stimulated LDs accumulation

**FIGURE 5:**
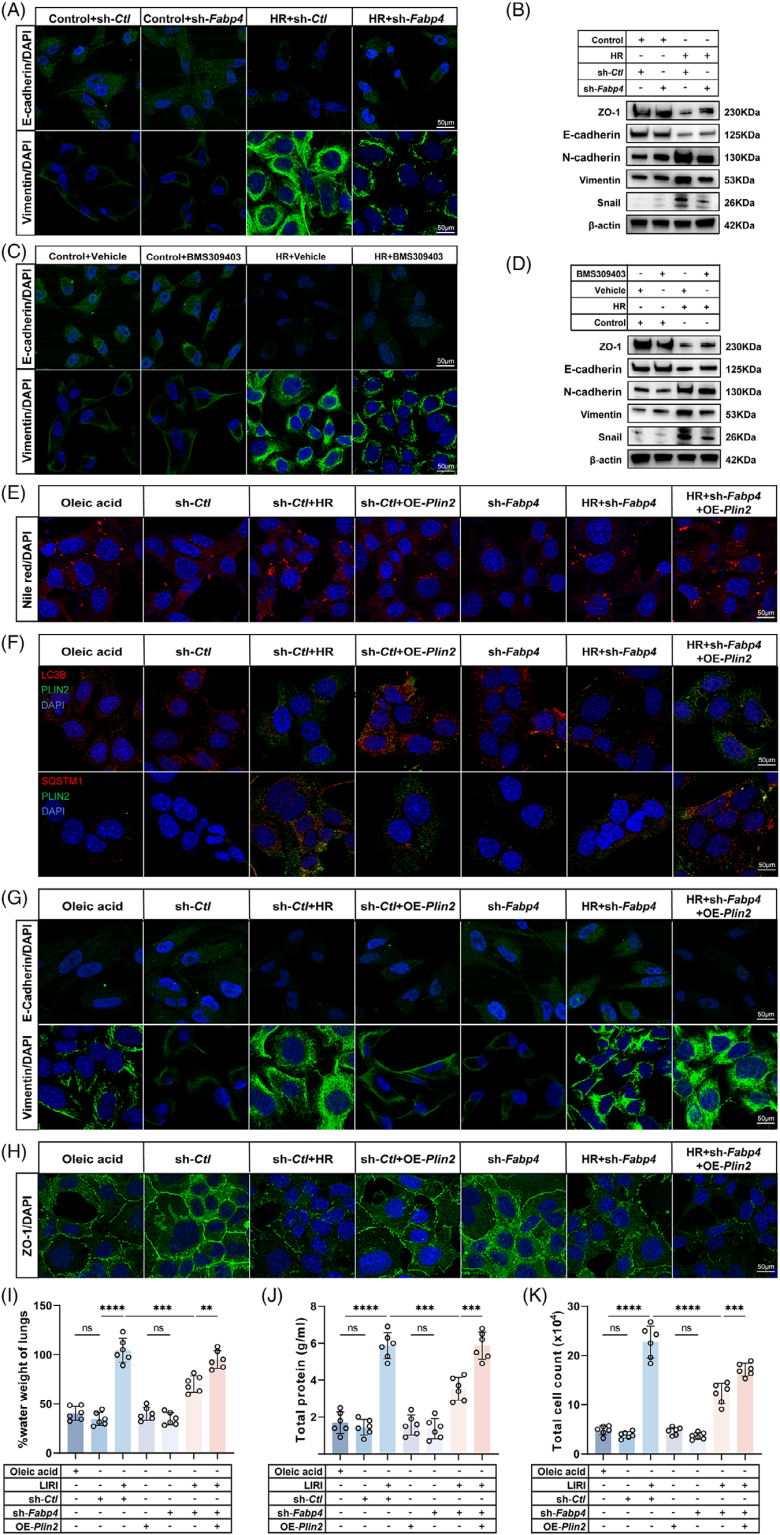
EMT is under the control of FABP4‐stimulated LDs accumulation. (A and B) MLE‐12 cells were infected with a FABP4‐knockdown lentivirus and a knockdown control lentivirus (sh‐*Ctl*). (A) Representative immunofluorescence images of E‐Cadherin (green) and Vimentin (green) (*n* = 5). Scale bars: 50 µm. (B) The protein expression of ZO‐1, E‐cadherin, N‐cadherin, Vimentin, Snail in MLE‐12 cells under HR, normalised to β‐actin (*n* = 5). (C and D) MLE‐12 cells were treated with FABP4 inhibitor BMS309403(10 µM). (C) Immunofluorescence images of E‐Cadherin (green) and Vimentin (green) (*n* = 5). Scale bars: 50 µm. (D) The protein expression of ZO‐1, E‐cadherin, N‐cadherin, Vimentin, Snail in MLE‐12 cells under HR, normalised to β‐actin (*n* = 5). (E–H) MLE‐12 cells were infected with sh‐*Ctl* or sh‐*Fabp4* lentivirus with or without PLIN2 overexpression (OE‐Plin2) and subjected to HR treatment. (E) Nile red staining in seven groups: Oleic acid (100 µM, 24 h), sh‐*Ctl*, sh‐*Ctl*+HR, sh‐*Ctl*+OE‐*Plin2*, sh‐*Fabp4*, HR+sh‐*Fabp4* and HR+sh‐*Fabp4*+OE‐*Plin2* (*n* = 5). Scale bars: 50 µm. (F) Immunofluorescence of LC3B, PLIN2 and SQSTM1 in the seven groups. Lipophagy was both visualised using LC3B and SQSTM1, and LDs were visualised using PLIN2. (*n* = 5). Scale bar: 50 µm. (G) Immunofluorescence images of E‐cadherin (green), vimentin (green) in the seven groups. (*n* = 5). Scale bars: 50 µm. (H) Immunofluorescence of ZO‐1 in the seven groups (*n* = 5). Scale bars: 50 µm. (I–K) In vivo validation using seven groups: Oleic acid (50 mg/kg, i.p.), AAV‐sh*Ctl*, AAV‐sh*Ctl*+LIRI, AAV‐sh*Ctl*+AAV‐*Plin2* OE, AAV‐sh*Fabp4*, AAV‐sh*Fabp4*+LIRI and AAV‐sh*Fabp4*+AAV‐*Plin2* OE+LIRI. (I) Lung wet/dry ratio (*n* = 6). (J) BALF total protein (*n* = 6). (K) BALF total cell count (*n* = 6). All data are presented as means ± SD. Statistical significance was determined by unpaired Student's *t*‐test or one‐way ANOVA followed by Tukey's post hoc test. ns: not significant, **p < .01, ***p < .001, ****p < .0001.

We next investigated whether LDs accumulation, driven by FABP4, is a prerequisite for AEB disruption under HR conditions. Given the known role of FABP4 in promoting EMT, we first assessed EMT hallmarks in MLE‐12 cells. HR exposure led to a significant decrease in the epithelial marker E‐cadherin and an increase in the mesenchymal marker vimentin (Figures 5A and S4C). We further confirmed EMT progression under HR by profiling a panel of markers: mesenchymal traits including N‐cadherin, vimentin, and Snail were up‐regulated, while epithelial markers such as ZO‐1 and E‐cadherin were down‐regulated (Figures 5B and S4D–H). Importantly, knockdown of FABP4 reversed the EMT phenotype in MLE‐12 cells under HR. Functionally, scratch‐wound assays showed that HR significantly enhanced cell migration, which was markedly suppressed by FABP4 knockdown (Figure S4A,B), confirming that FABP4‐mediated EMT promotes cellular motility. Pharmacological inhibition of FABP4 with BMS309403 corroborated these findings, reversing HR‐induced up‐regulation of mesenchymal markers (Figures 5C,D and S4I–O), thereby reinforcing the role of FABP4 in mediating EMT. To verify whether EMT directly drives barrier disruption, we treated MLE‐12 cells with ML‐327, a selective EMT inhibitor, prior to HR exposure. ML‐327 significantly preserved ZO‐1 expression and tight junction integrity (Figure S4P), demonstrating that EMT inhibition protects against barrier dysfunction.

In an attempt to establish a link between LDs accumulation and EMT, both being promoted by HR‐driven FABP4 signalling, we performed rescue experiments with comprehensive controls. MLE‐12 cells were infected with sh‐*Ctl* or sh‐*Fabp4* lentivirus, with or without PLIN2 overexpression, and subjected to HR treatment. HR induced robust LDs formation in sh‐*Ctl* cells, which was prevented by FABP4 knockdown. PLIN2 overexpression alone in sh‐*Ctl* cells without HR did not significantly increase LDs, indicating that PLIN2 requires lipid substrates to promote LDs formation. Importantly, PLIN2 overexpression in sh‐*Fabp4* cells restored LDs formation under HR conditions (Figures 5E and S4Q). Consistent with LDs restoration, PLIN2 overexpression in sh‐*Fabp4* cells under HR increased lipophagy impairment (Figures 5F and S4R), reactivated EMT markers (Figures 5G and S4S) and disrupted tight junction integrity (Figures 5H and S4T). This demonstrates that LDs accumulation downstream of FABP4 is sufficient to drive pathological epithelial remodelling. We hypothesised that increased LDs accumulation and EMT together contribute to AEB dysfunction. Using epithelial‐specific AAV‐shFabp4 and AAV‐*Plin2*‐OE vectors in mice, we demonstrated that AAV‐*Plin2*‐OE significantly exacerbated alveolar epithelial cell damage and lung permeability in AAV‐sh*Fabp4* mice (Figure S4U). Furthermore, AAV‐*Plin2*‐OE increased lung wet‐to‐dry ratio, BALF protein content and total cell count in AAV‐sh*Fabp4* mice (Figure 5I–K), supporting the functional importance of FABP4–LDs axis in promoting EMT and barrier disruption.

### 2.6 FABP4‐mediated LDs accumulation exacerbates long‐term lung injury and reduces survival in mice

**FIGURE 6:**
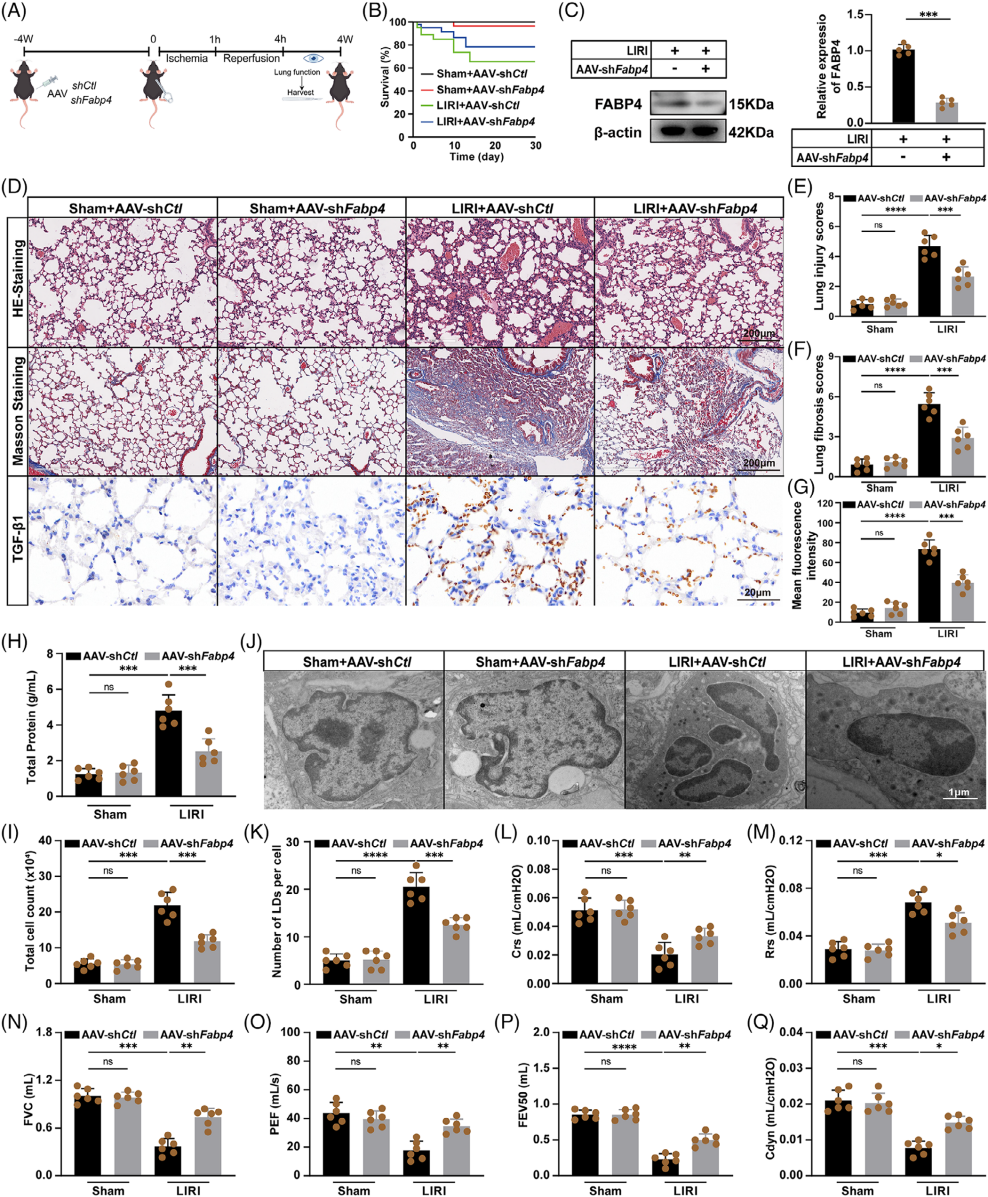
FABP4‐stimulated LDs accumulation exacerbates long‐term lung injury and survival in mice. A) Schematic illustration of the in vivo injection of FABP4‐knockdown AAV (sh*Fabp4*), and knockdown control AAV (sh*Ctl*) into mouse 4 weeks before LIRI. lungs were harvested on the last day of the observation period. (B) Kaplan–Meier survival curve of LIRI mice after injecting AAV‐sh*Fabp4* (*n* = 12 per group). (C) Western blot and quantification of FABP4 expression in lung tissues from AAV‐sh*Ctl* and AAV‐sh*Fabp4* mice 4 weeks post‐LIRI, normalised to β‐actin (*n* = 5). (D) Representative images of HE staining, Masson's trichrome staining, and TGF‐β immunohistochemical staining in lung sections from sham+AAV‐sh*Ctl*, sham+AAV‐sh*Fabp4*, LIRI+AAV‐sh*Ctl* and LIRI+AAV‐sh*Fabp4* mice. Scale bars: 200 µm for HE and Masson staining, 20 µm for TGF‐β staining. (E–G) Quantification of lung injury scores (E), collagen fibrosis scores (F) and TGF‐β mean fluorescence intensity (G) from panel D (*n* = 6). (H and I) Total protein content and cell count in BALF (*n* = 6). (J and K) Electron microscopy of LDs in mice lung (*n* = 6). Scale bars: 1 µm. (L–Q) Quantitative indices of lung function. (L) Crs, (M) Rrs, (N) FVC; (O) PEF; (P) FEV50; (Q) Cdyn (*n* = 6). Data were represented as means ± SD. Statistical significance was determined by unpaired Student's *t*‐test or one‐way ANOVA followed by Tukey's post hoc test. ns: not significant, *p < .05, **p < .01, ***p < .001, ****p < .0001.

We next evaluated whether FABP4 contributes to long‐term lung injury and mortality following LIRI, and whether its inhibition could preserve respiratory function. Epithelial‐specific knockdown of FABP4 was induced via AAV‐shFabp4 administration 4 weeks prior to LIRI, followed by a 4‐week observation period post‐injury (Figure 6A). AAV‐shFabp4 significantly improved survival in LIRI mice (78.5 vs. 65.4%; *n* = 12; Figure 6B), with sustained knockdown of FABP4 confirmed in lung tissues (Figure 6C). Histological analysis revealed preserved alveolar architecture and reduced inflammatory infiltration in AAV‐shFabp4‐treated mice, with significantly less collagen deposition on Masson's trichrome staining (Figure 6D–F). Immunohistochemical staining showed that LIRI increased TGF‐β expression in AAV‐shCtl mice, while AAV‐shFabp4 treatment significantly reduced TGF‐β levels (Figure 6D,G), indicating that FABP4 knockdown attenuates pulmonary fibrosis. BALF analysis showed reduced total protein and cellular content in the AAV‐shFabp4 group (Figure 6H,I), consistent with attenuated lung injury. Electron microscopy confirmed reduced and morphologically normal LDs in alveolar epithelial cells from AAV‐shFabp4 mice (Figure 6J,K).

Notably, lung function was markedly improved in the AAV‐shFabp4 group, with enhanced lung compliance (Crs), reduced airway resistance (Rrs), and improved respiratory parameters including forced vital capacity (FVC), peak expiratory flow (PEF), forced expiratory volume in 50 ms (FEV50) and dynamic compliance (Cdyn) (Figure 6L–Q). To further validate the therapeutic potential of FABP4 inhibition through pharmacological intervention, we administered the selective FABP4 inhibitor BMS309403 to mice prior to LIRI. Consistent with genetic knockdown results, BMS309403 treatment significantly improved survival rate (Figure S5C), preserved lung architecture, and reduced collagen deposition as demonstrated by HE and Masson staining (Figure S5A,B). These findings confirm that pharmacological inhibition of FABP4 recapitulates the protective effects observed with genetic knockdown, supporting the translational potential of FABP4 inhibitors for treating LIRI‐associated lung injury. Collectively, these findings demonstrate that targeting FABP4 provides sustained protection against long‐term pulmonary dysfunction following LIRI.

### 2.7 Circulating FABP4 correlates with ARDS in CPB patients

**FIGURE 7:**
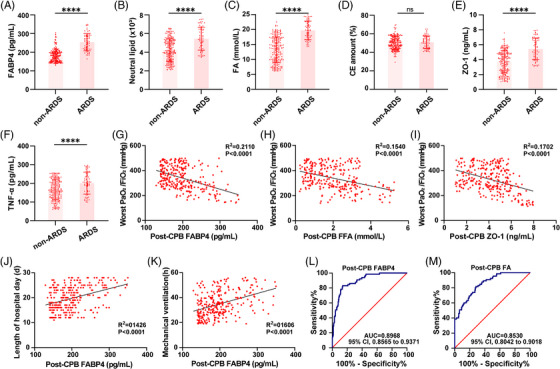
Circulating FABP4 correlates with ARDS in patients after CPB. (A–F) Serum levels of FABP4, neutral lipid, FA, CE, ZO‐1, TNF‐α in non‐ARDS (*n* = 215) and ARDS patients (*n* = 65). (G) Correlation between serum FABP4 levels and worst PaO_2_/FiO_2_ levels in all patients. *n* = 280. (H) Correlation between serum FA levels and worst PaO_2_/FiO_2_ levels in all patients. *n* = 280. (I) Correlation between serum ZO‐1 levels and worst PaO_2_/FiO_2_ levels in all patients. *n* = 280. (J and K) Spearman correlation analysis between post‐CPB FABP4 levels and length of hospital stay (J) or mechanical ventilation duration (K) (*n* = 280). (L and M) Receiver‐operating characteristic curve (ROC) analysis for the prediction of ARDS after CPB by using post‐operative FABP4 level and post‐operative FA level. Data were represented as means ± SD. Statistical significance was determined by Student's *t*‐test or Spearman correlation for correlation analyses. ns: not significant, ****p < .0001.

**FIGURE 8:**
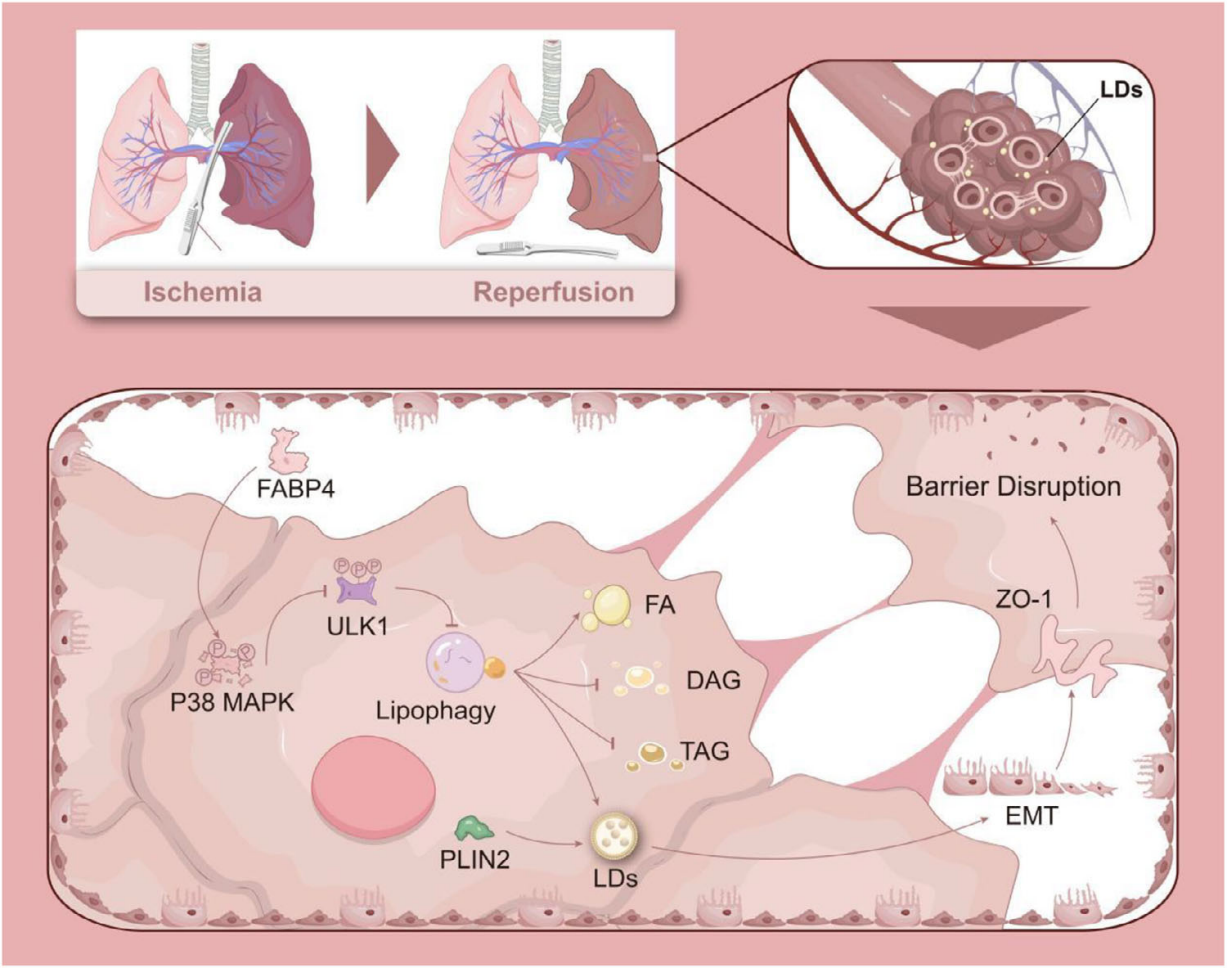
FABP4‐mediated LDs accumulation drives AEB disruption in LIRI.

To evaluate the clinical relevance of our findings, we analysed a cohort of 280 patients undergoing cardiopulmonary bypass (CPB), including 65 who developed ARDS and 215 who did not. Serum FABP4 levels were significantly elevated in the ARDS group (Figure 7A). Consistent with our preclinical models, serum lipidomic profiling revealed increases in FAs and neutral lipids, but not CEs, in ARDS patients (Figure 7B–D). Markers of alveolar epithelial injury, including serum zonula occludens‐1 (ZO‐1) and tumor necrosis factor‐α (TNF‐α), were significantly higher in the ARDS group (Figure 7E,F), indicating greater epithelial damage. Multivariable logistic regression analysis adjusting for age, body mass index (BMI) and lipid parameters confirmed that FABP4 remained an independent predictor of ARDS (odds ratio [OR] = 1.062, 95% confidence interval [CI]: 1.037–1.087, *p* < .001) (Table S2). Spearman's correlation analysis revealed strong inverse associations between the ratio of arterial oxygen partial pressure to fractional inspired oxygen (PaO_2_/FiO_2_) and FABP4, FFA and ZO‐1 levels (Figure 7G–I). Moreover, elevated serum FABP4 levels were significantly correlated with extended length of hospital stay and prolonged mechanical ventilation duration (Figure 7J,K), indicating that FABP4 not only predicts ARDS occurrence but also reflects disease severity and clinical outcomes. Receiver operating characteristic (ROC) analysis showed that both FABP4 and FA were strong predictors of CPB‐associated ARDS, with area under the curve values of.897 (95% CI,.857–.937) and.853 (95% CI,.804–.902), respectively (Figure 7L,M). Collectively, these data identify FABP4 as a critical mediator of CPB‐induced ARDS and support its potential as a predictive biomarker and therapeutic target in perioperative lung injury. A schematic summary of the proposed mechanism is presented in Figure 8.

## 3 DISCUSSION

Emerging evidence has shown that EMT is closely associated with alveolar injury and repair in various lung diseases.^28^, ^29^ In line with previous studies,^30^, ^31^ our work confirms that HR induces EMT in murine alveolar epithelial MLE‐12 cells, as evidenced by the loss of epithelial adherens junctions, down‐regulation of the epithelial marker E‐cadherin, and up‐regulation of the mesenchymal marker Vimentin. While EMT is well recognised in pulmonary fibrosis, it is also partially initiated during acute lung injury. Notably, partial EMT may facilitate reversion to the epithelial phenotype, promoting tissue repair.^32^ This phenotypic plasticity highlights the complexity of EMT in LIRI and suggests a context‐dependent function shaped by the alveolar microenvironment. We propose that lung ischaemia initiates a transient mesenchymal shift in epithelial cells to promote motility and repair. However, when reperfusion injury follows, this adaptive response may become maladaptive, enhancing mesenchymal transition and contributing to fibrosis and barrier dysfunction. Our findings identify LDs accumulation, driven by LIRI, as a key mediator of this phenotypic transition toward a mesenchymal‐like, pro‐fibrotic epithelial state.

We demonstrate that FABP4 is a central regulator of lipid‐based metabolic reprogramming in alveolar epithelial cells during LIRI. While previous studies have linked lipid metabolism to EMT,^33^, ^34^ our work specifically addresses the role of this axis in ischaemia–reperfusion injury. We previously reported that endothelial cells under HR conditions shift from oxidative phosphorylation to glycolysis.^35^ Here, we extend this concept to epithelial cells, showing that HR promotes FABP4‐dependent LDs accumulation, which supports energy demands and contributes to AEB disruption. Mechanistically, we reveal that FABP4 induces LDs accumulation by activating p38 MAPK signalling, which in turn induces ULK1 phosphorylation (Ser757), thereby suppressing lipophagy. This distinguishes LIRI from other LD‐inducing insults, such as infection,^36^ where β‐oxidation of FFAs from LDs contributes to inflammation. In the hypoxic environment of LIRI, epithelial cells accumulate FFAs in LDs to mitigate lipotoxicity, yet following reoxygenation, these stored lipids become fuel for the mesenchymal transition and AEB breakdown.

Our findings further clarify the role of the FABP4–MAPK/ULK1–lipophagy axis in LIRI. Genetic knockdown of FABP4 reduced both LDs accumulation and inflammatory cytokine release,^37^ underscoring its importance in epithelial lipid metabolism. This is consistent with evidence that FABP4 promotes lipid‐induced EMT in other contexts, such as colon cancer.^38^ We further documented the MAPK/ULK1/lipophagy pathway wherein ULK1 phosphorylation is stimulated by p38 MAPK. Remarkably, this autocrine FABP4 activation in epithelial cells differs from that described in models where chronic lipolysis leads to FABP4 secretion^39^ or where adipocyte‐derived FABP4 promotes JNK/c‐Jun signalling to disrupt blood–brain barrier.^40^ Of note, FABP4 was reported to shift cell metabolism from glucose uptake to increased intracellular lipid accumulation.^41^ Whether, LDs accumulation were increased leading to EMT and AEB disruption was, however, not addressed. We demonstrate that only FABP4, among FABP isoforms, drives LDs accumulation and barrier dysfunction in our ischaemia–reperfusion model. This supports a model in which LIRI triggers an autocrine FABP4‐dependent signalling cascade that promotes both mesenchymal transition and LDs formation, facilitating AEB disruption.

While FABP4 was identified as an attractive target to protect barrier injury resulting from tight junction degradation^40^, ^42^, ^43^, ^44^ and encouraging survival results have been reported in infection^45^, ^46^ and aging‐associated metabolic disorders,^47^ our results provide strong rationale for targeting FABP4 in the context of lung injury. Potential therapeutic target may also be derived from the observation that LDs represent a distinct metabolic signature to support airway inflammation.^48^ We show that pharmacological inhibition or genetic silencing of FABP4 limits LDs formation and EMT, thereby preserving epithelial integrity under HR stress. This suggests that FABP4 inhibitors, some of which are already under clinical investigation, may be repurposed for treating ARDS or CPB‐associated lung injury. Importantly, AAV‐mediated FABP4 knockdown significantly improved survival in our LIRI mouse model. Our findings also align with previous reports linking LDs accumulation to barrier dysfunction in other tissues, such as the choroid plexus.^49^ EMT has also been implicated in epithelial barrier disruption in asthma,^50^ further supporting our conclusion that LDs play a critical role in EMT and barrier breakdown. These observations open the door to new therapeutic approaches aimed at preserving epithelial function by limiting lipid accumulation and mesenchymal transition. Finally, our data suggest that ULK1 phosphorylation is a key downstream event of FABP4 signalling, consistent with prior studies linking ULK1 to EMT and autophagy.^51^, ^52^ While those studies did not focus on the epithelial barrier, we demonstrate that FABP4 deletion protects against both LDs accumulation and AEB disruption in HR‐treated epithelial cells. This positions FABP4 as a particularly promising target for preventing CPB‐induced lung injury.

In conclusion, we identify an autocrine FABP4‐EMT axis as a key mediator of lipid metabolic adaptation and barrier dysfunction in alveolar epithelial cells during LIRI. By elucidating this pathway, our study not only expands understanding of the molecular mechanisms underlying lung injury but also highlights novel therapeutic targets for reducing AEB disruption and improving outcomes in CPB‐associated ARDS and lung injury.

## 4 LIMITATION OF THE STUDY

While we validated FABP4 in HSAEpiC and clinical samples (*n* = 280), direct examination of patient lung tissue was not available. Additionally, although MLE‐12 is a well‐established model, validation in human primary alveolar cells would be valuable. Finally, the precise upstream regulators of FABP4—hypoxia‐inducible factors, inflammatory cytokines or metabolic stress—warrant further investigation. Regarding therapeutic translation, while BMS309403 showed efficacy in our preclinical model, challenges including limited bioavailability, short half‐life and potential systemic metabolic effects must be addressed for clinical development. Nevertheless, our comprehensive approach provides strong translational evidence for the FABP4–LDs–EMT axis.

## 5 MATERIALS AND METHODS

### 5.1 Human study design and ethics

Between April 2023 and December 2024, we enrolled 280 consecutive patients (aged 41–85 years) undergoing cardiac surgery with CPB at The First Affiliated Hospital with Nanjing Medical University (Nanjing, China). The institutional review board approved this protocol (No. 2023‐SR‐513, 23 March, 2023), which followed Declaration of Helsinki principles. Informed consent was obtained from each participant.

Ineligible patients included those undergoing off‐pump surgery or having: existing lung pathology, prior cardiac surgery, cardiomyopathy, congenital heart disease, acute heart failure, inflammatory/autoimmune disorders, infections, malignancy, transplant history, corticosteroid treatment or investigator affiliations.

Clinical data were extracted from electronic records. Radial arterial blood samples were collected before surgery and on post‐operative day 1. After centrifugation, samples were frozen at −80°C. We measured serum FABP4 and TNF‐α using ELISA kits (CUSABIO) and ZO‐1 using ELISA kits (Westang Bio‐Tech).

### 5.2 Animal model of LIRI

Seven‐week‐old male C57BL/6J mice (22 ± 3 g; Zhejiang Vital River Laboratory Animal Technology, Beijing, China) were used. Under sodium pentobarbital anaesthesia (40 mg/kg i.p.), animals underwent tracheotomy and mechanical ventilation (1 mL tidal volume, 150 breaths/min; HUAYON, Shenzhen) on a warming pad. Through left thoracotomy, we occluded the left hilum using an atraumatic clamp for 60 min, then allowed reperfusion for 180 min. Following pulmonary function assessment (BUXCO, DSI, USA), blood was collected from the vena cava and harvested left lung tissue.

Animals were randomised using computer‐generated sequences. Two blinded investigators independently evaluated lung injury from coded slides, with unblinding performed post‐scoring.

The Nanjing Medical University Animal Care and Use Committee approved all procedures (IACUC‐2404068), conducted per institutional and NIH guidelines.

### 5.3 AAV administration

To achieve epithelial‐specific overexpression or knockdown of PLIN2, and knockdown of FABP4, the following AAV vectors were used: AAV‐SPC‐sh*Ctl* (negative control), AAV‐SPC‐sh*Plin2*, AAV‐SPC‐*Ctl*‐OE (negative control), AAV‐SPC‐*Plin2*‐OE and AAV‐SPC‐sh*Fabp4* (GeneChem, China). Vectors were suspended in sterile saline for tail vein delivery (2.5 × 10⁰ vg/mouse).

### 5.4 Histopathological analysis

Lung tissues underwent formalin fixation, paraffin embedding and sectioning (5 µm). H&E‐stained sections were examined under light microscopy (Nikon Ni‐U, Japan), and we quantified inflammation using the Szapiel scoring system. For fibrosis assessment, Masson's trichrome staining (AWI0253; Abiowell, China) were performed following standard protocols: sequential application of haematoxylin, acid fuchsin, phosphomolybdic acid and aniline blue, with ethanol dehydration and xylene clearing before mounting.

### 5.5 BALF analysis

Following LIRI, BALF samples were collected and centrifuged at 1500×*g* for 15 min. We quantified total protein using a BCA assay kit (P0012S; Beyotime, China) and determined cell counts with an automated counter (Countstar, China).

### 5.6 Evans Blue dye extravasation assay

Evans Blue (1%, 30 mg/kg; MB4680‐1; MeilunBio, China) was injected intravenously at reperfusion onset. Following PBS perfusion to remove intravascular dye, left lungs were collected, dried (72°C, 24 h), weighed, and incubated in formamide (HY‐Y0842; MCE, USA) at 37°C for 24 h. Extracted dye was quantified spectrophotometrically at 620 nm.

### 5.7 Lung oedema assessment

Lung oedema severity was assessed via wet‐to‐dry (W/D) weight ratio determination. Post‐euthanasia pulmonary specimens were weighed fresh (wet weight), followed by thermal desiccation (60°C, 48 h) to constant weight (dry weight). The resulting W/D ratio quantified oedema extent.

### 5.8 Oil Red O staining

The left lung lobe underwent 4% PFA fixation (overnight, 4°C), Following triplicate PBS washing cycles (20 min each), specimens received optimal cutting temperature (OCT) compound embedding after tissue immersion. Cryostat‐generated 10‐µm sections (CM1850; Leica, Germany) were maintained at −80°C pending staining procedures. Oil Red O staining followed manufacturer protocols (Cat. No. C0157M; Beyotime), with imaging performed via Nikon Eclipse Ti inverted microscopy platform (Japan).

### 5.9 Transmission electron microscopy

Ultrastructural analysis employed transmission electron microscopy following standard preparation protocols. Post‐LIRI pulmonary tissues underwent harvest, tryptic digestion and low‐speed centrifugal pelleting (225×*g*, 5 min). Cell pellets received dual fixation: primary fixation with 4% glutaraldehyde (2 h, 4°C) followed by secondary fixation with 1% osmium tetroxide (1 h, 4°C). Specimens then underwent graded dehydration via ethanol/acetone series before epoxy resin embedding. Ultrathin sections received uranyl acetate and lead citrate contrast staining prior to examination via JEM‐1400Plus transmission electron microscope (JEOL Ltd., Tokyo, Japan).

### 5.10 Lipidomics analysis

Comprehensive lipid profiling employed targeted metabolomics approaches. Harvested pulmonary specimens underwent lipidomic characterisation with raw metabolite acquisition via UPLC‐MS/MS utilising Q300 Kit reagents (Metabo‐Profile, Shanghai, China), followed by computational processing through iMAP platform. Supplementary lipid analysis utilised Waters ACQUITY UPLC system interfaced with Waters XEVO TQ‐S mass spectrometry platform, with data analysis performed via MassLynx 4.1 software (Waters, Milford, MA, USA). Statistical identification of differential lipid species employed Student's *t*‐test (significance thresholds: *p* < .05 and |log2FC| ≥ 1). Metabolic pathway enrichment analysis leveraged Small Molecule Pathway Database (SMPDB) resources.

### 5.11 Folch extraction methodology facilitated total lipid recovery from tissue and serum

specimens. Serum CE quantification employed Amplex™ Red Cholesterol Assay Kit (Thermo Fisher Scientific #A12216) following manufacturer specifications. FA compositional analysis involved saponification procedures (.5 M KOH/methanol, 70°C, 1 h) succeeded by methylation reactions (14% BF_3_‐methanol, 70°C, 30 min). Resulting FA methyl esters underwent hexane‐based extraction and GC‐MS characterisation utilising DB‐23 capillary column (dimensions: 60 m × .25 mm).

### 5.12 RNA isolation and RNA‐seq analysis

Total RNA extraction from lung specimens was accomplished using TRIzol reagent (Invitrogen, USA). RNA integrity and concentration were verified using an Agilent 2100 Bioanalyzer (Agilent Technologies, USA). Library construction and sequencing were performed on the DNBSEQ platform (BGI Co., Ltd., Shenzhen, China). DEGs comparing LIRI versus sham groups were identified using the limma R package with threshold criteria of *p* < .05 and |log2FC| > 1.

### 5.13 scRNA‐seq sample preparation

Single‐cell suspension generation followed established protocols. Freshly excised pulmonary specimens underwent dual washing with ice‐cold RPMI‐1640 medium supplemented with.04% BSA under aseptic conditions. Following mincing into approximately.5 mm^3^ pieces, tissues underwent enzymatic disaggregation at 37°C (30–60 min duration) with periodic gentle agitation at 5–10 min intervals. Cell suspensions were filtered via 40 µm BD strainers, followed by centrifugal pelleting (300×*g*, 5 min). Erythrocyte depletion utilised RBC lysis buffer treatment (MACS Cat. 130‐094‐183; 100 µL volume, 10 min, 4°C). Following PBS washing and centrifugation, cellular pellets underwent resuspension in 100 µL medium. Luna counter technology assessed viability and concentration parameters. Concentration adjustment to 700–1200 cells/µL preceded single‐cell capture procedures and library construction using 10× Genomics Chromium Next GEM Single Cell 3′ Reagent Kit v3.1 (MACS Cat. 1000268). Illumina NovaSeq 6000 PE150 platform performed sequencing operations.

### 5.14 ScRNA‐seq data analysis

Sequencing data preprocessing employed fastp (v0.23.1) for adapter trimming and quality control. Following alignment to mouse reference genome via Cell Ranger pipeline (v6.1.2), gene expression matrices were generated. Downstream computational analysis utilised R environment (v4.3.1) with Seurat package (v4.4.0). Quality control retained cells meeting criteria: > 500 detected genes and <10% mitochondrial transcript content. Normalisation procedures (NormalizeData function) preceded identification of 3000 highly variable features (FindVariableFeatures). Post‐scaling, principal component analysis was performed, followed by Harmony‐based (v1.2.0) batch effect correction. Dimensionality reduction via t‐SNE employed the leading 10 Harmony dimensions, with clustering performed at.1 resolution.

### 5.15 Differential expression and enrichment analysis

DEGs between LIRI and sham groups were identified using *Seurat*’s *FindMarkers* function, with thresholds of adjusted *p* value < .05 and |log2FC| > .5. Functional enrichment was performed using GSEA with the *fgsea* R package (v1.28.0), as well as Gene Ontology and KEGG pathway analysis using the *clusterProfiler* package (v4.10.1).

### 5.16 Cell culture and treatment

MLE‐12 cells (Fenghbio, Changsha, China) were maintained in DMEM (Gibco, USA) enriched with 10% heat‐inactivated foetal bovine serum, l‐glutamine, penicillin and streptomycin. Cultures were maintained at 37°C in a humidified incubator with 5% CO_2_ atmosphere. HSAEpiC (Fenghbio) were cultured in Airway Epithelial Cell Medium (Fenghbio) according to the manufacturer's instructions. To simulate HR injury, cells were incubated in glucose‐free D‐Hank's solution under 1% O_2_/94% N_2_/5% CO_2_ for 4 h, followed by reoxygenation for 4 h. In selected experiments, cells were treated with: p38 MAPK inhibitor SB202190 (10 µM; MCE; Cat. No. HY‐10295), p38 MAPK activator anisomycin (10 µM; Santa Cruz, USA; Cat. No. sc‐3524A), FABP4 inhibitor BMS309403 (10 µM; MCE; Cat. No. HY‐101903), FABP4 recombinant protein (100 ng/mL; MCE; Cat. No. HY‐P75215), triacsin C (5 µM, MCE; Cat. No. HY‐N6707), ML‐327(5 µM; MCE; Cat. No. HY‐103038), Oleic acid (100 µM; MCE; Cat. No. HY‐N1446).

### 5.17 Lentiviral transduction

Lentiviral constructs for PLIN2 knockdown/overexpression and FABP4 knockdown/overexpression, along with their respective controls, were obtained from GeneChem (Shanghai, China). MLE‐12 cells at 80% confluence were incubated with 500 µL of lentivirus‐containing basal medium for 2 h. The medium was replaced with fresh DMEM, and cells were cultured for an additional 48 h before being harvested for qRT‐PCR and Western blot analysis.

### 5.18 Immunofluorescence

Cell sections were used for immunofluorescent staining with the following primary antibodies: PLIN2, LC3B, P62, E‐cadherin (1:100; Proteintech, China; vimentin, and ZO‐1 (details in Table S3). The secondary antibodies included Alexa Fluor 488 goat anti‐rabbit and Alexa Fluor 568 goat anti‐mouse.

MLE‐12 cells cultured on poly‐l‐lysine‐coated glass coverslips underwent 4% PFA fixation (10 min), PBS washing and blocking via 10% goat serum with.3% Triton X‐100 (Biosharp BS084) for 30 min at ambient temperature. Primary antibody exposure occurred overnight at 4°C, succeeded by secondary antibody treatment (2 h, room temperature). Nuclear visualisation employed DAPI Fluoromount‐G counterstaining (SouthernBiotech 0100–20, 1:1000 dilution, 5 min). Confocal laser scanning microscopy (Leica) captured images at optimal wavelengths. Experimental replication (minimum five iterations) included analysis of five randomly selected fields per specimen.

### 5.19 Scratch‐wound migration assay

MLE‐12 cells were plated in 6‐well culture plates at 5 × 10⁵ cells per well and grown to 90–95% confluency. Following lentiviral transduction (sh‐*Ctl* or sh‐*Fabp4*, 48 h) and serum deprivation (.5% FBS, 12 h), a uniform scratch was introduced using a sterile 200 µL pipette tip. After PBS washing and medium replacement with serum‐free formulation, cells underwent HR treatment or were maintained under normoxic conditions. Phase‐contrast imaging was performed at 0 and 24 h post‐scratch using a Nikon Eclipse Ti inverted microscope (Japan).

### 5.20 Nile Red staining

MLE‐12 cells were seeded into confocal dishes and subjected to HR treatment. Cells were fixed in 4% PFA for 10 min, followed by staining with Nile Red (Meilun, China) for 20 min and DAPI Fluoromount‐G for 5 min at room temperature. Fluorescent images were captured using a confocal laser scanning microscope.

### 5.21 TEER and FITC–dextran permeability assay

Post‐HR treatment, MLE‐12 cells received culture on transwell insert platforms (Corning 3460). Epithelial resistance measurements employed voltohmmeter technology (World Precision Instruments, USA), with electrode configuration positioning: long electrode in basolateral chamber, short electrode in apical compartment. Epithelial permeability was assessed using a FITC–dextran leakage assay (Millipore, Billerica, MA). After a 1‐h FITC–dextran incubation, fluorescence intensity of the leaked tracer was visualised with confocal microscopy (excitation 488 nm, emission 525 nm).

### 5.22 Immunohistochemistry

For immunohistochemical analysis, tissue sections underwent overnight incubation at 4°C with anti‐TGF‐β1 antibodies (complete details in Table S3). After thorough washing, sections were exposed to appropriate secondary antibodies. Chromogenic development employed 3,3′‐diaminobenzidine substrate. Haematoxylin counterstaining was subsequently performed to reveal tissue architecture.

### 5.23 Western blotting and Co‐IP

Lung tissues and cultured cells were lysed in buffer containing protease inhibitors. Protein quantification employed BCA methodology (Beyotime Kit P0012S), with subsequent thermal denaturation at 100°C for 10 min. Equivalent protein quantities underwent electrophoretic separation via 10% SDS‐PAGE, followed by transfer to PVDF membranes and blocking procedures. Horizontal membrane sectioning preceded primary antibody exposure, encompassing anti‐FABP4, p38, p‐P38, JNK, p‐JNK, ERK, p‐ERK, ULK1, p‐ULK1, PLIN1‐5, ZO‐1, occludin, claudin‐3, LC3, P62, N‐cadherin, E‐cadherin, vimentin, Snail and β‐actin (details in Table S3). HRP‐conjugated secondary antibodies were used, and signals were visualised by ECL (MeilunBio; Cat. No. MA0186) and quantified using ImageJ (NIH, USA). Membranes were stripped (Beyotime; P0025B) and re‐probed as necessary. For Co‐IP, Protein lysates (500 µg) in RIPA buffer with protease and phosphatase inhibitors (Beyotime; P1005 and P1046) were pre‐cleared with Protein A/G magnetic beads (Bimake, B23202) for 1 h at 4°C. Supernatants were incubated with 2 µg of primary antibodies (anti‐FABP4 or anti‐p38) or IgG controls (Beyotime; A7016 or A7028) overnight at 4°C, followed by incubation with 30 µL Protein A/G beads for 2 h at 4°C. Bead‐bound complexes were washed four times with IP wash buffer (50 mM Tris‐HCl pH 7.4, 150 mM NaCl,.1% NP‐40), eluted with 2× SDS loading buffer at 100°C for 10 min, and analysed by Western blot using anti‐p38 or anti‐FABP4 antibodies to detect co‐immunoprecipitated proteins.

### 5.24 Quantitative reverse transcription PCR

RNA isolation employed RNeasy Mini Kit methodology (Qiagen, Hamburg, Germany). Reverse transcription of 2 µg RNA generated complementary DNA templates. Quantitative PCR amplification proceeded through 40 cycles with thermal profile: 95°C (5 s) and 60°C (30 s). Relative expression quantification utilised the 2^−ΔΔCT calculation method. Primer sequences alongside PCR parameters are detailed in Table S4.

### 5.25 Statistical analysis

Statistical presentation employed mean ± standard deviation or median with interquartile range based on data distribution characteristics. Two‐group comparative analyses utilised unpaired Student's *t*‐test, while multiple group assessments employed one‐way analysis of variance coupled with Tukey's multiple comparison post‐hoc testing. Survival trajectory analysis implemented log‐rank (Mantel–Cox) methodology. Association analyses employed Spearman's rank correlation coefficient. Categorical data underwent *χ* ^2^ test evaluation. Alpha threshold for statistical significance was established at *p* < .05. Computational analyses utilised IBM SPSS Statistics v27 (IBM Inc.) and GraphPad Prism 9 software (GraphPad Inc.).

## AUTHOR CONTRIBUTIONS

The contributions of each author are as follows. Peng Lu: designed experiments. Xiaoning Lu: designed experiments. Zihao Shen: performed the experiments and wrote the manuscript, contributed equally to this work. Yuanpu Qi: performed the experiments and wrote the manuscript, contributed equally to this work. Mingyu Chu: performed the experiments and wrote the manuscript, contributed equally to this work. Minchao Wu: carried out the animal experiments, contributed equally to this work. Chen Feng: carried out the animal experiments. Xiangyu Li: carried out the animal experiments. Zhaoyang Liu: carried out the animal experiments. Linjie Si: carried out the animal experiments, Yongliang Wang: carried out the animal experiments.

## CONFLICT OF INTEREST STATEMENT

The authors declare no conflicts of interest.

## ETHICS STATEMENT

Research involving human material was obtained from the Institutional Review Board (Protocol No. 2023‐SR‐513, approved on 23 March, 2023). Animal experiments were approved by the Animal Care and Use Committee of Nanjing Medical University (IACUC‐2404068).

## Supporting information



Supporting Information

## Data Availability

The data that support the findings of this study are available from the corresponding author upon reasonable request.
