# Supplementary material for: FABP4‐mediated lipid droplet accumulation drives epithelial–mesenchymal transition and aggravates alveolar epithelial barrier disruption
Source: Clin Transl Med. 2025 Dec 26;16(1):e70563. doi: 10.1002/ctm2.70563 (PMC12743144; doi:10.1002/ctm2.70563)

**FABP4-mediated Lipid Droplet Accumulation Drives Epithelial-Mesenchymal Transition and Aggravates Alveolar Epithelial Barrier Disruption**

Zihao Shen^a*^, Yuanpu Qi^a*^, Mingyu Chu^a*^, Minchao Wu^a^, Chen Feng^b^, Xiangyu Li^a^, Zhaoyang Liua, Linjie Si^a^, Yongliang Wang^b^, Jialin Zhang^a^, Xiaoning Lu^b‡^ and Peng Lu^a‡^

^a^Department of Cardiovascular Surgery, The First Affiliated Hospital with Nanjing Medical University, Nanjing, Jiangsu 210029, China; ^b^Department of Cardiothoracic Surgery, The Affiliated Suqian First People’s Hospital of Nanjing Medical University, Suqian, Jiangsu 223800, China.

^‡^**Correspondence** should be addressed to Peng Lu, Department of Cardiovascular Surgery, The First Affiliated Hospital with Nanjing Medical University, Nanjing, Jiangsu 210029, China. E-mail: penglu@njmu.edu.cn.

ORCID ID: 0000-0002-3263-7765 (P.L.).

^*^These authors contributed equally to this work.

**ABBREVIATIONS** AEB: alveolar epithelial barrier; ARDS: acute respiratory distress syndrome; BALF: bronchoalveolar lavage fluid; CE: cholesteryl ester; CPB: cardiopulmonary bypass; EMT: epithelial-mesenchymal transition; FA: fatty acid; FABP4: fatty acid-binding protein 4; FFA: free fatty acid; HR: hypoxia/reoxygenation; LDs: lipid droplets; LIRI: Lung ischemia/reperfusion injury; TEER: Trans-epithelial electrical resistance; TG: triglyceride; ZO-1: zonula occludens-1

Table of Contents

**TABLE S1** …………………………………………………………………………………3

**TABLE S2** …………………………………………………………………………………4

**TABLE S3** …………………………………………………………………………………5

**TABLE S4** …………………………………………………………………………………6

**FIGURE S1**…………………………………………………………………………………7

**FIGURE S2**…………………………………………………………………………………8

**FIGURE S3**…………………………………………………………………………………10

**FIGURE S4**…………………………………………………………………………………12

**FIGURE S5**…………………………………………………………………………………14

**Table S1. Differential analysis of lipid metabolites**

| Class | Metabolite | P_val | FDR | log2FC | OPLSDA_VIP |
| --- | --- | --- | --- | --- | --- |
| Cer | Cer(d18:1\|20:2) | 0.011268611 | 0.105039718 | -0.243377446 | 1.377658599 |
| Cer | Cer(d18:1\|22:2) | 0.024023051 | 0.158906553 | -0.205317156 | 1.479685206 |
| Cer | Cer(d18:1\|22:6) | 0.008658009 | 0.101417226 | 0.557826371 | 1.502208456 |
| DAG | DAG(14:0\|18:2) | 0.039559715 | 0.210985146 | 0.171952683 | 1.26637629 |
| DAG | DAG(16:0\|20:1) | 0.009860049 | 0.102080511 | -0.088859931 | 1.579265155 |
| DAG | DAG(16:1\|18:2) | 0.029768442 | 0.171778553 | 0.15565087 | 1.007203646 |
| HexCer | HexCer(d18:1\|14:1) | 0.01230396 | 0.105039718 | -0.352789059 | 1.656041909 |
| HexCer | HexCer(d18:1\|16:1) | 0.029556577 | 0.171778553 | 0.257157839 | 1.418854865 |
| HexCer | HexCer(d18:1\|18:1) | 0.000346474 | 0.009381444 | 0.39358439 | 1.929152229 |
| HexCer | HexCer(d18:1\|22:3) | 0.025974026 | 0.160401003 | 0.244367112 | 1.114746927 |
| LPC | LPC 16:0 | 0.002426129 | 0.047444305 | -0.127802728 | 1.880712915 |
| LPC | LPC 16:1 | 0.008658009 | 0.101417226 | -0.709455125 | 1.64208781 |
| LPC | LPC 18:0 | 0.008712947 | 0.101417226 | -0.087904299 | 1.806555845 |
| LPC | LPC 20:0 | 0.000287378 | 0.008429755 | -0.709810005 | 1.817758088 |
| LPC | LPC(22:1) | 0.000538482 | 0.013538979 | -0.334618817 | 1.914480937 |
| LPC | LPC 24:0 | 0.024139616 | 0.158906553 | -0.216875416 | 1.593786087 |
| LPC | LPC(O-18:0) | 0.014669483 | 0.120085069 | -0.41655214 | 1.498754605 |
| LPC | LPC(P-18:1) | 0.0383222 | 0.207529452 | -0.156119202 | 1.340771046 |
| LPE | LPE(14:0) | 0.02437771 | 0.158906553 | -0.309294623 | 1.567411461 |
| LPE | LPE 20:0 | 0.001407185 | 0.033021938 | -0.381608201 | 1.797745319 |
| LPE | LPE(20:5) | 0.009130953 | 0.101417226 | -0.395741666 | 1.655355021 |
| LPE | LPE 22:5 | 0.004835619 | 0.072669633 | -0.253997192 | 1.764632293 |
| PC | PC(16:0e\|18:0) | 0.012533148 | 0.105039718 | 0.259573001 | 1.691261959 |
| PC | PC(16:0e\|18:2) | 5.14049E-05 | 0.003015751 | 0.215445154 | 2.093135749 |
| PC | PC(16:0e\|20:1) | 0.00315974 | 0.055611424 | 0.46588914 | 1.84572642 |
| PC | PC(16:0e\|20:2) | 0.011967947 | 0.105039718 | 0.348606823 | 1.659392789 |
| PC | PC(16:0p\|16:1) | 0.015151515 | 0.121212121 | -0.334229547 | 1.169140002 |
| PC | PC(16:0p\|18:1) | 1.05141E-05 | 0.002538703 | 0.214757693 | 2.150496454 |
| PC | PC(16:0p\|18:3) | 0.044864959 | 0.229556142 | -0.123075753 | 1.305908962 |
| PC | PC(16:0p\|20:0) | 0.00315974 | 0.055611424 | 0.46588914 | 1.84572642 |
| PC | PC(16:0p\|20:1) | 0.01214524 | 0.105039718 | 0.351987666 | 1.699255306 |
| PC | PC(16:0p\|20:2) | 0.00013178 | 0.006626664 | 0.316428514 | 2.045878125 |
| PC | PC(18:0e\|16:0) | 0.012533148 | 0.105039718 | 0.259573001 | 1.691261959 |
| PC | PC(18:0e\|18:1) | 0.004954748 | 0.072669633 | 0.453665014 | 1.799443353 |
| PC | PC(18:0e\|18:2) | 0.010967757 | 0.105039718 | 0.365077546 | 1.666861618 |
| PC | PC(18:0e\|18:3) | 0.000253921 | 0.008429755 | 0.267814689 | 2.011779167 |
| PC | PC(18:0p\|14:1) | 0.004329004 | 0.071863077 | -0.360852883 | 1.209830076 |
| PC | PC(18:0p\|16:0) | 0.028873898 | 0.171778553 | 0.040241549 | 1.349466716 |
| PC | PC(18:0p\|16:1) | 3.19188E-05 | 0.002538703 | 0.218354011 | 2.115437628 |
| PC | PC(18:0p\|18:0) | 0.009733772 | 0.102080511 | 0.367139594 | 1.73149263 |
| PC | PC(18:0p\|18:1) | 0.027954223 | 0.169653215 | 0.318547622 | 1.567109939 |
| PC | PC(18:0p\|18:2) | 0.000279033 | 0.008429755 | 0.282379451 | 2.002839873 |
| PC | PC(18:0p\|18:3) | 0.007080457 | 0.092308178 | 0.061460738 | 1.603704216 |
| PC | PC(18:1e\|16:1) | 2.88044E-05 | 0.002538703 | 0.210914009 | 2.120810936 |
| PC | PC(18:1e\|18:0) | 0.009219748 | 0.101417226 | 0.415758783 | 1.729972889 |
| PC | PC(18:1e\|18:1) | 0.005478209 | 0.074166518 | 0.377253578 | 1.799707658 |
| PC | PC(18:1e\|18:2) | 0.000230481 | 0.008429755 | 0.29361148 | 2.003390748 |
| PC | PC(18:2e\|16:0) | 2.88044E-05 | 0.002538703 | 0.210914009 | 2.120810936 |
| PC | PC(18:2e\|18:0) | 0.005266899 | 0.074157934 | 0.383533168 | 1.822618487 |
| PC | PC(18:2e\|18:1) | 0.000271043 | 0.008429755 | 0.262909954 | 2.012949395 |
| PC | PC(18:2p\|18:0) | 3.60611E-05 | 0.002538703 | 0.294086281 | 2.095427421 |
| SM | SM(d18:1\|16:0) | 0.017330642 | 0.129795447 | -0.077920084 | 1.660518528 |
| SM | SM(d18:1\|18:4) | 0.002164502 | 0.044817927 | -0.33663329 | 1.809087348 |
| SM | SM(d18:1\|20:0) | 0.011828241 | 0.105039718 | -0.108858188 | 1.577599718 |
| SM | SM(d18:1\|22:1) | 0.023450922 | 0.158906553 | -0.119136365 | 1.5607995 |
| FFA | FFA(12:0) | 0.016090643 | 0.123128397 | 0.727101991 | 1.388444056 |
| FFA | FFA(14:1) | 0.025208901 | 0.160401003 | 1.240933348 | 1.440896938 |
| FFA | FFA(16:2) | 0.025974026 | 0.160401003 | 0.435233756 | 1.892989327 |
| FFA | FFA(18:2) | 0.020651205 | 0.15144217 | 0.08770994 | 1.479518367 |
| PE | PE(16:0\|16:1) | 0.023100262 | 0.158906553 | -0.334479862 | 1.522411666 |
| PE | PE(16:0\|20:3) | 0.044998221 | 0.229556142 | -0.124097344 | 1.2623035 |
| PE | PE(16:1\|16:1) | 0.015657582 | 0.12247709 | -0.44372066 | 1.492320471 |
| PE | PE(16:1\|18:0) | 0.002164502 | 0.044817927 | -0.152032255 | 1.707254276 |
| PE | PE(16:1\|18:1) | 0.004491442 | 0.071863077 | -0.300783092 | 1.661900172 |
| PE | PE(18:0\|20:3) | 0.023693216 | 0.158906553 | -0.147843405 | 1.589790193 |
| PE | PE(18:0\|22:5) | 0.034432208 | 0.192383132 | -0.191155943 | 1.518748697 |
| PI | PI(16:0\|20:3) | 0.041125541 | 0.216062544 | -0.28628811 | 1.308283736 |
| PI | PI(18:1\|18:1) | 0.031444186 | 0.178521827 | 0.521620675 | 1.48394444 |

**Table S2. Multivariable Logistic Regression Analysis for Predictors of ARDS after CPB**

| Variable | OR (95% CI) | P Value |
| --- | --- | --- |
| Triglycerides (per mmol/L) | 0.745 (0.401–1.386) | 0.35 |
| Total cholesterol (per mmol/L) | 1.414 (0.723–2.766) | 0.31 |
| Age (per year) | 1.124 (1.035–1.222) | 0.006* |
| Body mass index (per kg/m²) | 0.984 (0.790–1.226) | 0.89 |
| FABP4 (per ng/mL) | 1.062 (1.037–1.087) | <0.001*** |
| Diabetes mellitus | 1.778 (0.750–4.216) | 0.19 |

Definition of abbreviations: CI = confidence interval; FABP4 = fatty acid-binding protein 4; OR = odds ratio. **P* <0.05, ****P* <0.001

**Table S3. Primary antibodies used in westernblotting, immunohistochemistry and immunofluorescence**

| Antibody | Application | Species | Dilution | Company | Catalog Number |
| --- | --- | --- | --- | --- | --- |
| FABP4 | WB | Rabbit | 1:1000 | Proteintech | 12802-1-AP |
| p38 | WB | Rabbit | 1:1000 | Proteintech | 14064-1-AP |
| p-p38 | WB | Rabbit | 1:1000 | Proteintech | 28796-1-AP |
| JNK | WB | Rabbit | 1:1000 | Proteintech | 51153-1-AP |
| p-JNK | WB | Rabbit | 1:1000 | Proteintech | 80024-1-RR |
| ERK | WB | Rabbit | 1:1000 | Santa Cruz | sc-81459 |
| p-ERK | WB | Rabbit | 1:1000 | Santa Cruz | sc-7383 |
| ULK1 | WB | Rabbit | 1:1000 | Abcam | ab177472 |
| p-ULK1 (Ser757) | WB | Rabbit | 1:1000 | Abcam | ab229909 |
| PLIN1 | WB | Rabbit | 1:1000 | Proteintech | 27716-1-AP |
| PLIN2 | WB | Rabbit | 1:1000 | Proteintech | 15294-1-AP |
| PLIN2 | IF | Rabbit | 1：100 | Proteintech | 15294-1-AP |
| PLIN3 | WB | Rabbit | 1:1000 | Proteintech | 10694-1-AP |
| PLIN5 | WB | Rabbit | 1:1000 | Proteintech | 26951-1-AP |
| ZO-1 | WB | Rabbit | 1:1000 | Proteintech | 21773-1-AP |
| ZO-1 | IF | Rabbit | 1：100 | Proteintech | 21773-1-AP |
| Occludin | WB | Rabbit | 1:1000 | Proteintech | 27260-1-AP |
| Claudin-3 | WB | Rabbit | 1:1000 | Proteintech | 86697-1-RR |
| LC3 | WB | Rabbit | 1:1000 | Proteintech | 14600-1-AP |
| LC3 | IF | Mouse | 1：100 | ABclonal | A17424 |
| P62/SQSTM1 | WB | Rabbit | 1:1000 | Proteintech | 18420-1-AP |
| N-cadherin | WB | Rabbit | 1:1000 | Proteintech | 22018-1-AP |
| E-cadherin | WB | Rabbit | 1:1000 | Proteintech | 20874-1-AP |
| E-cadherin | IF | Rabbit | 1：100 | Proteintech | 20874-1-AP |
| Vimentin | WB | Rabbit | 1:1000 | Abcam | ab92547 |
| Vimentin | IF | Rabbit | 1：100 | Abcam | ab92547 |
| Snail | WB | Rabbit | 1:1000 | Abcam | ab180714 |
| β-actin | WB | Rabbit | 1:10000 | Proteintech | 20536-1-AP |
| TGF-β1 | IHC | Rabbit | 1:200 | Proteintech | 21898-1-AP |

Definition of abbreviations: FABP4 , Fatty acid-binding protein 4; p38, p38 mitogen-activated protein kinase; p-p38, Phosphorylated p38; JNK, c-Jun N-terminal kinase; p-JNK, Phosphorylated JNK; ERK, Extracellular signal-regulated kinase; p-ERK, Phosphorylated ERK; ULK1, Unc-51 like autophagy activating kinase 1; p-ULK1, Phosphorylated ULK1 (Ser757); PLIN1/2/3/5, Perilipin 1/2/3/5; ZO-1, Zonula occludens-1; LC3, Microtubule-associated protein 1 light chain 3; P62/SQSTM1, Sequestosome 1; TGF-β1, Transforming growth factor beta 1. WB, Western blot; IHC, Immunohistochemistry; IF, Immunofluorescence.

**Table S4. Primer sequences**

| Gene | Forward(5'-3') | Reverse(5'-3') |
| --- | --- | --- |
| FABP4 | AAGGTGAAGAGCATCATAACCCT | TCACGCCTTTCATAACACATTCC |
| PLIN1 | CTGTGTGCAATGCCTATGAGA | CTGGAGGGTATTGAAGAGCCG |
| PLIN2 | CTTGTGTCCTCCGCTTATGTC | GCAGAGGTCACGGTCTTCAC |
| PLIN5 | CTTCCTGCCCATGACTGAGG | GACCCCAGACGCACAAAGTAG |
| β-Actin | GGCATCCTGACCCTGAAGTAC | GATCTTCATGAGGTAGTCTGTC |

**Figure S1. Alveolar Epithelial Cells Accumulate Lipid Droplets in LIRI.** A) The mRNA expression of PLIN1, PLIN2, PLIN3, PLIN5 in MLE-12 cells under HR (n=5). All mRNA levels were measured by qPCR, normalized to β-Actin. B) Western blot analysis of PLIN2 protein expression normalized to that of β-Actin after MLE-12 cells were infected with PLIN2-knockdown lentivirus (n=5). C) Western blot validation of two independent shRNA sequences targeting PLIN2 (AAV-sh*Plin2*#1 and AAV-*shPlin2*#2) in mouse lung tissue, showing effective knockdown compared to control (AAV-sh*Ctl*) (n=5). D-F) Mice were injected with PLIN2-kncokdown AAV (sh*Plin2*) and knockdown control AAV (sh*Ctl*). D) Lung wet/dry weight ratio (n=6). E, F) Total protein content and cell count in BALF (n=6). All data are presented as means ± SD. Statistical significance was determined by Student's t-test or one-way ANOVA followed by Tukey's post hoc test. *ns*: not significant, **p* < 0.05, ***p* < 0.01, ****p* < 0.001, *****p* < 0.0001.


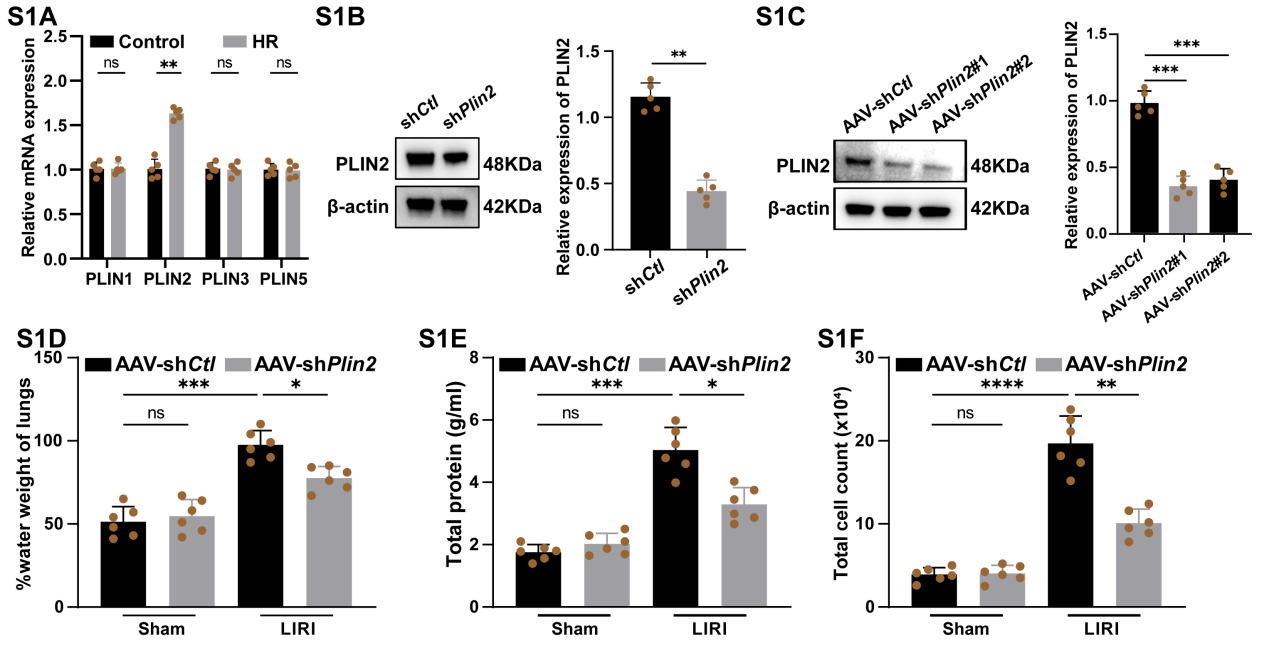


**Figure S2. FABP4 Signaling Supports LDs Formation and Alveolar Epithelial Barrier Disruption.** A) Volcano plot of differentially expressed genes (DEGs) in epithelial cells between LIRI and Sham groups. B) The mRNA expression of FABP4 in MLE-12 cells under HR and in mice under LIRI (n=5). All mRNA levels were measured by qPCR, normalized to β-Actin. C) Quantification of FABP4 protein levels in mice under LIRI, normalized to β-Actin (n=5). D) Quantification of FABP4 protein levels in MLE-12 cells under HR conditions, normalized to β-Actin (n=5). E) Quantification of FABP4 protein levels in HSAEpiC under HR conditions, normalized to β-Actin (n=5). F) Secreted FABP4 levels in culture media of MLE-12 cells under

HR conditions, measured by ELISA (n=5). G) Time-course analysis of FABP4 protein levels in mouse lung tissue at different time points after reperfusion (0h, 1h, 3h, 6h, 12h, 24h). H) Western blot validation of FABP4 knockdown in MLE-12 cells

transduced with sh-*Fabp4* or sh-*Ctl* lentivirus, and quantification normalized to β-Actin (n=5). I) qPCR validation of FABP4 knockdown efficiency in mouse lung tissue treated with AAV-sh*Fabp4* normalized to β-Actin (n=5). J) Western blot validation of two independent shRNA sequences targeting FABP4 (AAV-sh*Fabp4*#1 and AAV-sh*Fabp4*#2) in mouse lung tissue, demonstrating effective knockdown compared to control (AAV-sh*Ctl*) (n=5). K) IL-6 levels in lung tissue measured by ELISA (n=6). L) TNF-α levels in lung tissue measured by ELISA (n=6). M, N) MLE-12 cells were treated with P38 agonist Anisomycin and infected with FABP4-knockdown lentivirus. M) Nile red staining of MLE-12 cells under HR (n=5). Scale bars=50 µm. N) Tight junctions were visualized by staining for ZO-1 (n=5). Scale bars=50 µm. All data are presented as means ± SD. Statistical significance was determined by unpaired Student's t-test or one-way ANOVA followed by Tukey's post hoc test. *ns*: not significant, ***p* < 0.01, ****p* < 0.001, *****p* < 0.0001.


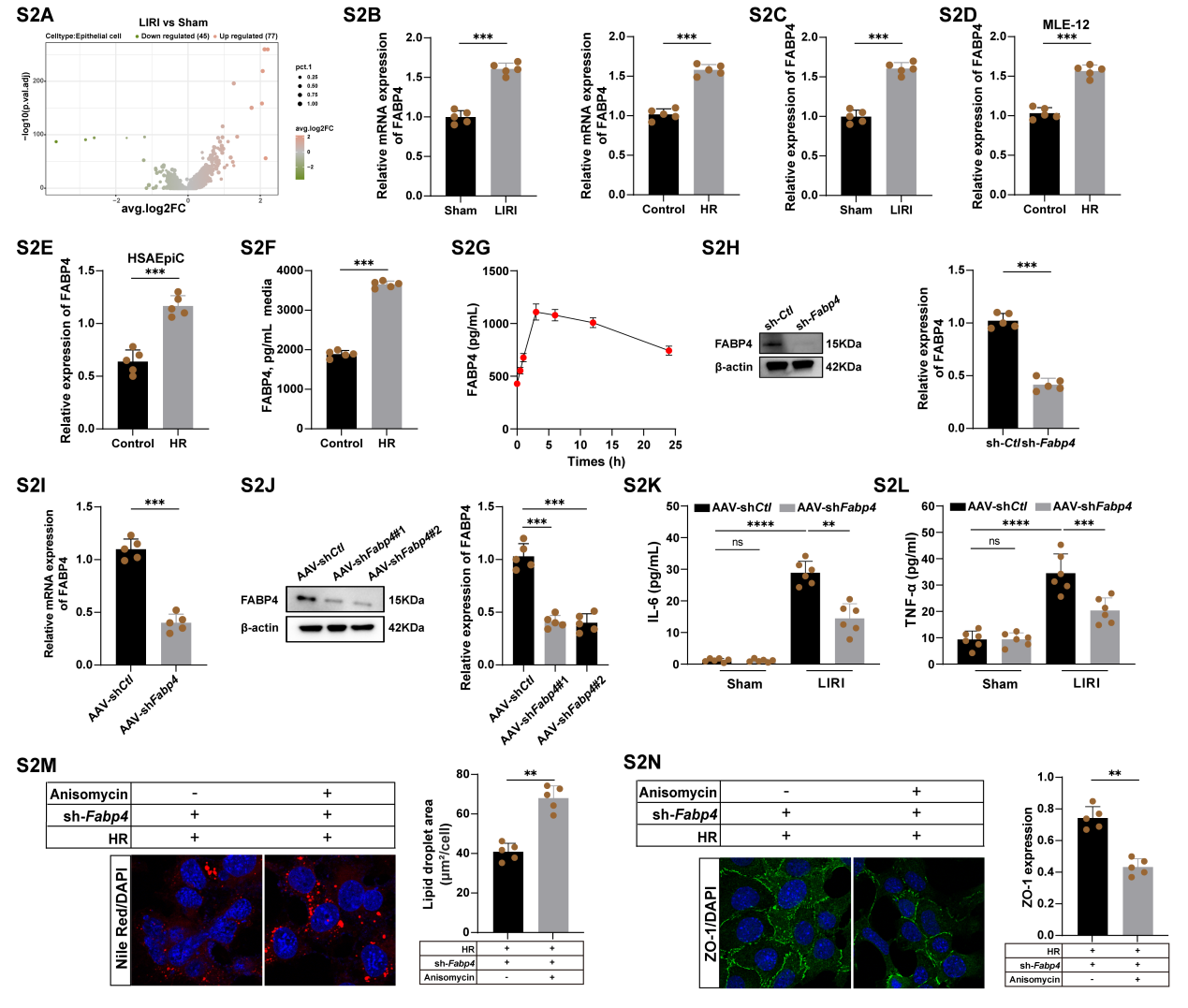


**Figure S3. FABP4-mediated Lipophagy Promotes FA Metabolism and TG Formation.** A) Volcano plot of differential lipid metabolites between LIRI and Sham groups. B) Serum FA in mice under LIRI (n=6). C-E) Quantification of p-p38/p38 ratio, p-ERK/ERK ratio and p-JNK/JNK ratio in LIRI lung tissues from AAV-sh*Ctl* and AAV-sh*Fabp4* mice (n=5). F) Co-IP showing direct interaction between FABP4 and p38 MAPK in MLE-12 cells under HR (n=5). G-I) Lung wet/dry ratio (G), BALF protein (H), and BALF cell count (I) in AAV-sh*Fabp4* mice treated with Anisomycin under LIRI (n=6). J, K) Quantification of p-ULK1 Ser757/ULK1 in LIRI tissues (J) and HR-treated cells (K) (n=5). L, M) Western blot and quantification of p-p38/p38 and p-ULK1 Ser757/ULK1 in sh-Fabp4 cells treated with recombinant FABP4 (100 ng/mL) under HR (n=5). N, O) Confocal images of MLE-12 cells treated with P38 inhibitor SB202190 under HR. Lipophagy was both visualized using LC3B and SQSTM1, and LDs were visualized using PLIN2. The number of LC3B puncta, SQSTM1 puncta and PLIN2 puncta were quantified. (n=5). Scale bar: 50μm. P) Quantification of LC3-II/LC3-I and p62 in MLE-12 cells infected with sh-*Fabp4* under HR (n=5). Q) Western blot of LC3 and p62 in cells treated with Triacsin C (5 μM) under HR (n=5). R, S) Quantification of p-ULK1 Ser757/ULK1 (R) and lipid droplets (S) in MLE-12 cells treated with SB202190 under HR (n=5). All data are presented as means ± SD. Statistical significance was determined by unpaired Student's t-test (B,C-E, F, J, K, L, M, P, Q, R, S) or one-way ANOVA followed by Tukey's post hoc test (G-I, N, O). *ns*: not significant, ***p* < 0.01, ****p* < 0.001, *****p* < 0.0001.


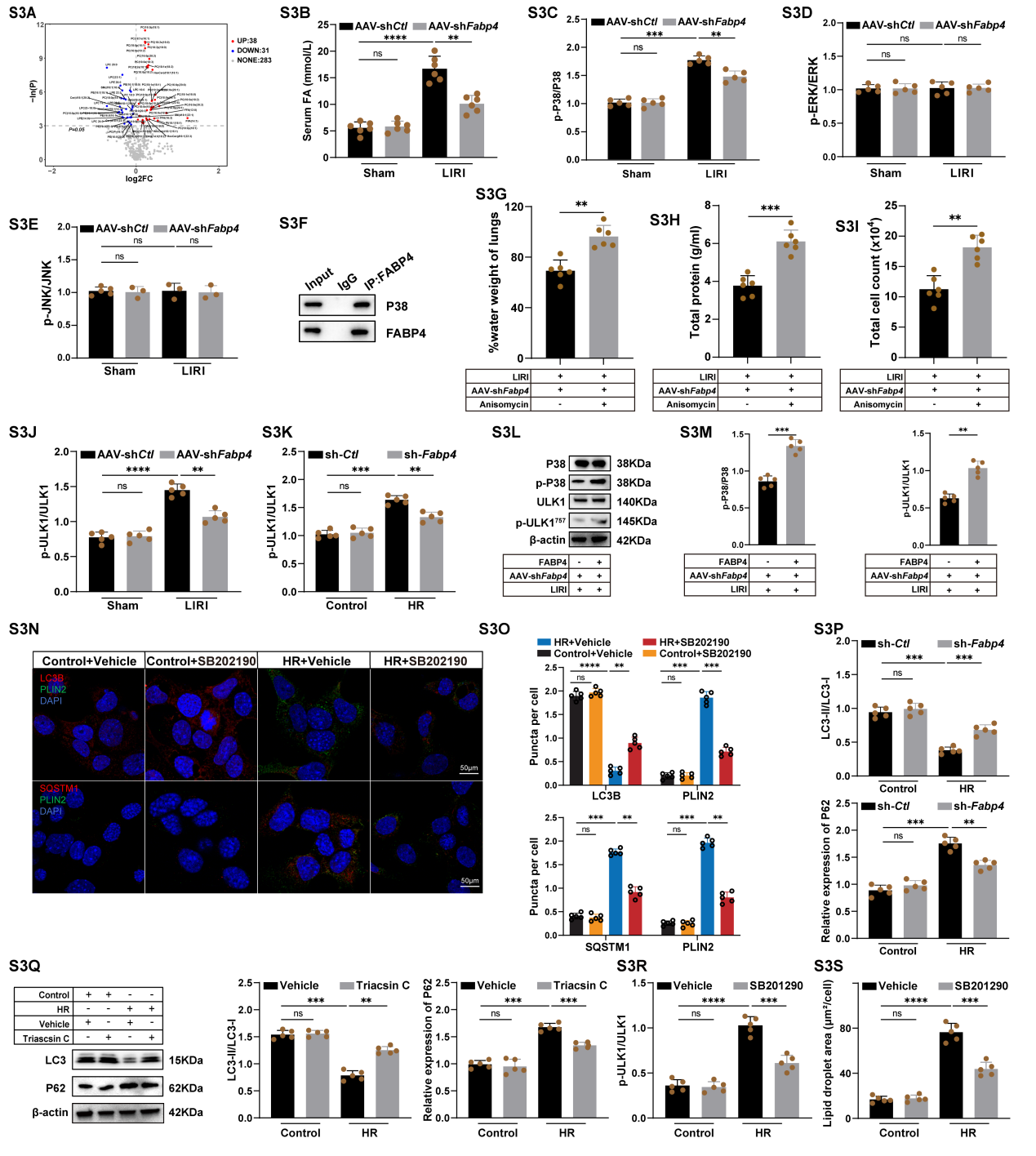


**Figure S4. EMT is Under the Control of FABP4-stimulated LD Accumulation.** A, B) Scratch-wound assay images (A) and quantification of migration distance (B) in MLE-12 cells under Control, Control+sh-*Fabp4,* HR, and HR+sh-*Fabp4* conditions (n=5). C) Quantification of E-cadherin and Vimentin fluorescence intensity from Figure 5A (n=5). D-H) Quantification of Western blot from Figure 5B: ZO-1 (D), E-cadherin (E), N-cadherin (F), Vimentin (G), and Snail (H) (n=5). I-O) Quantification of E-cadherin and Vimentin fluorescence intensity from Figure 5C in MLE-12 cells treated with BMS309403 under Control or HR conditions (n=5). P) Immunofluorescence of ZO-1 in MLE-12 cells treated with ML-327 (5 μM) or vehicle under Control or HR conditions (n=3). Scale bars: 50 μm. Q) Quantification of Nile red staining intensity from Figure 5E (n=5). R) Quantification of LC3B ,SQSTM1, and PLIN2 puncta from Figure 5F (n=5). S) Quantification of E-cadherin and Vimentin fluorescence intensity from Figure 5G (n=5). T) Quantification of ZO-1 fluorescence intensity from Figure 5H (n=5). U) HE staining of lung sections showing alveolar epithelial damage in AAV-shFabp4 mice with or without AAV-Plin2-OE under LIRI, with lung injury scores (n=6). Scale bars: 200 μm.

All data are presented as means ± SD. Statistical significance was determined by unpaired Student's t-test or one-way ANOVA followed by Tukey's post hoc test (A-U). *ns*: not significant, **p* < 0.05, ***p* < 0.01, ****p* < 0.001, *****p* < 0.0001.


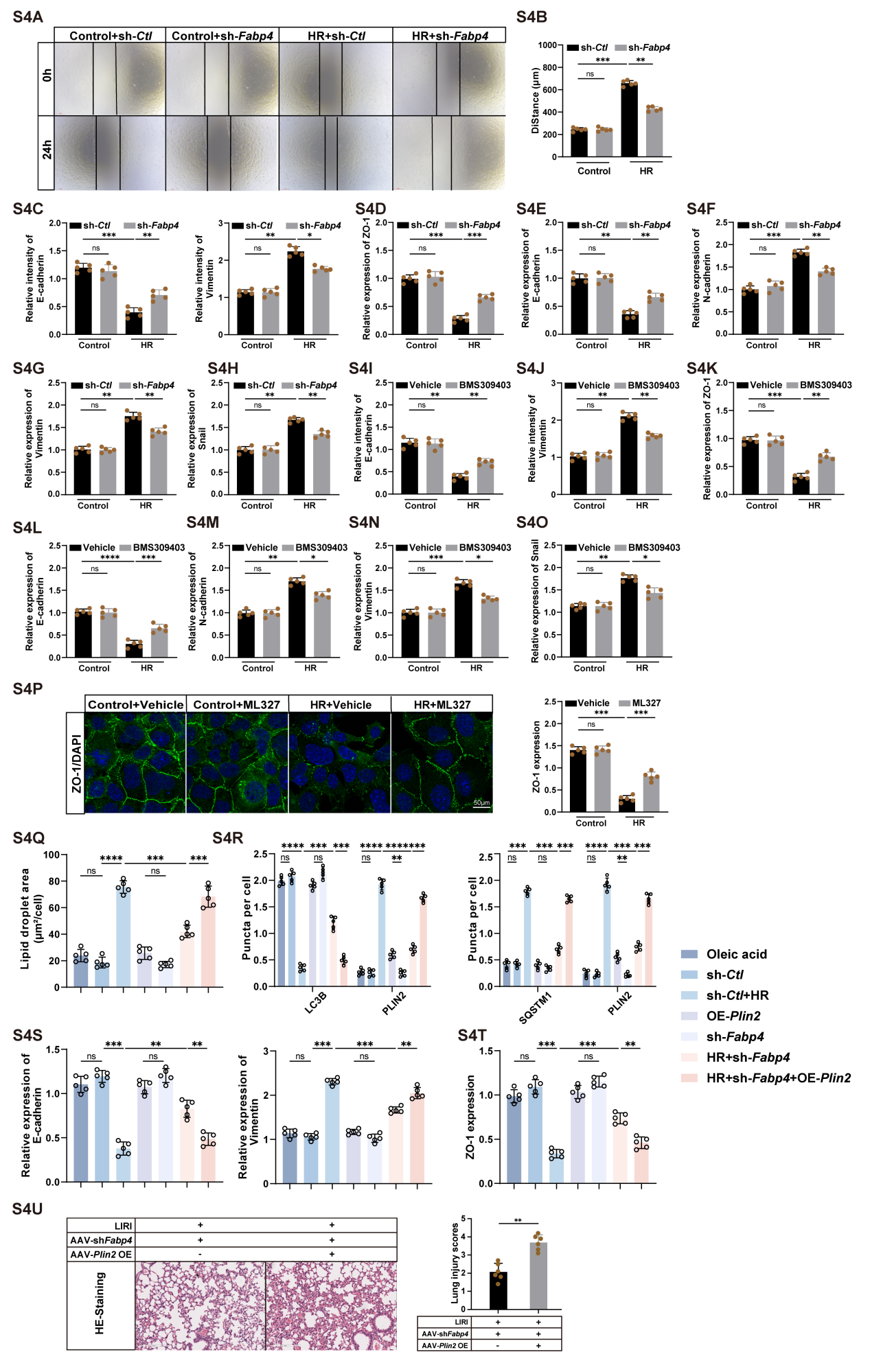


**Figure S5. Pharmacological Inhibition of FABP4 Improves Long-term Outcomes Following LIRI.**

A) HE staining and Masson's trichrome staining of lung sections from mice treated with BMS309034 or vehicle following LIRI, with quantification of lung injury scores and lung fibrosis scores (n=6). Scale bars: 200 μm. B) Kaplan-Meier survival curves of Sham+Vehicle, Sham+BMS309034, LIRI+Vehicle, and LIRI+BMS309034 mice (n=12 per group). All data are presented as means ± SD. Statistical significance was determined by log-rank test for survival analysis or one-way ANOVA with Tukey's post-hoc test for other comparisons. *ns*: not significant, ***p* < 0.01, ****p* < 0.001, *****p* < 0.0001.
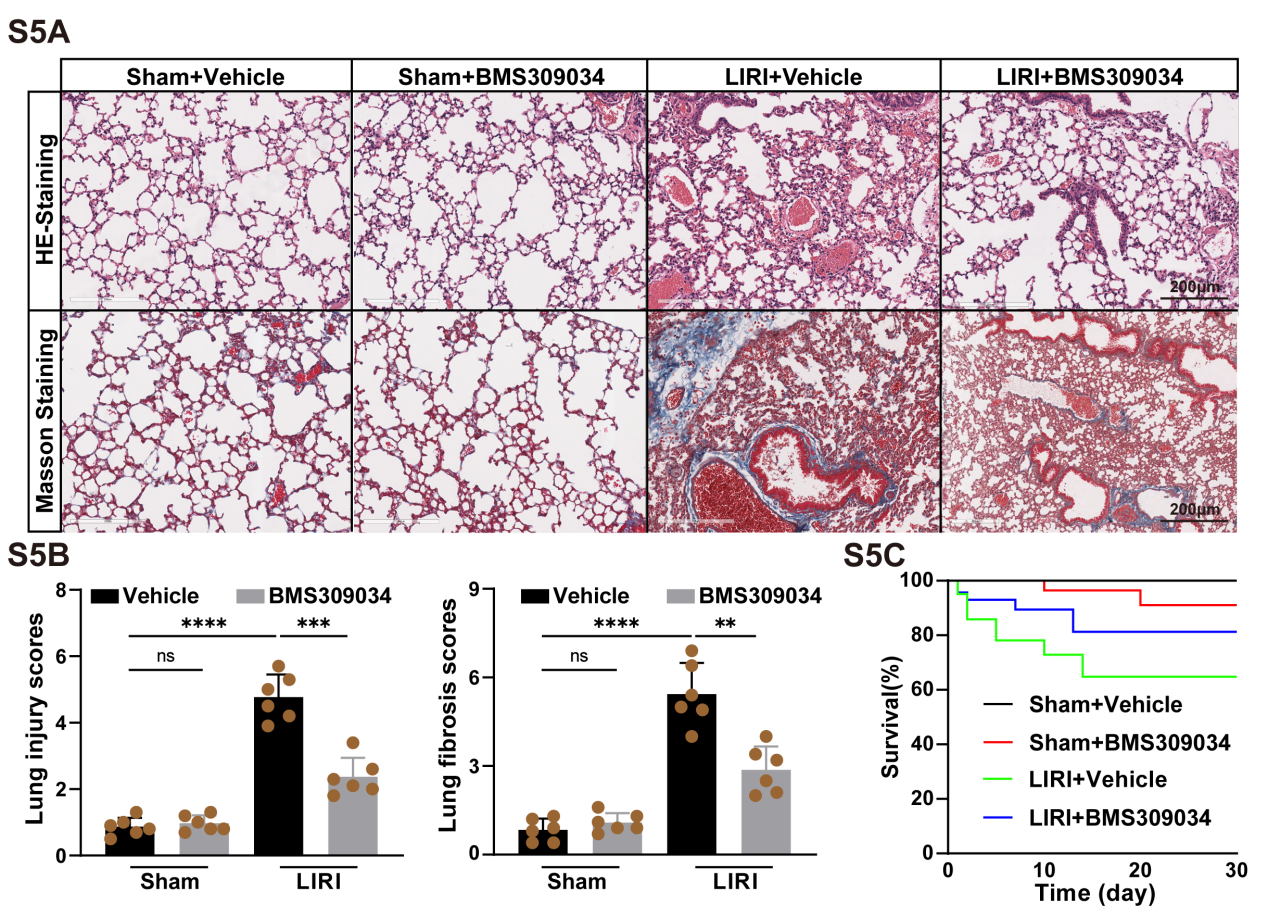

Supplement: Supplementary file 1 — Supporting Information [file CTM2-16-e70563-s001.docx]
